# NO and NO_2_ reactions with oxygenated peroxy radicals lead to indistinguishable product compositions: computational insights from cyclohexene oxidation in presence of NO_*x*_

**DOI:** 10.1039/d5ea00151j

**Published:** 2026-07-14

**Authors:** Sakshi Jha, Avinash Kumar, Prasenjit Seal, Siddharth Iyer, Matti Rissanen

**Affiliations:** a Aerosol Physics Laboratory, Tampere University Tampere 33720 Finland sakshi.jha@tuni.fi sakshijha1498@gmail.com; b Department of Chemistry, University of Helsinki Helsinki 00560 Finland

## Abstract

This study presents a computational investigation into the role of nitrogen oxides (NO_*x*_) in modulating gas-phase cyclohexene oxidation by ozone (O_3_) and hydroxyl radical (OH) through their reactions with peroxy radicals (RO_2_) formed during the oxidation sequence. While NO_2_ suppresses further oxidation by forming peroxynitrates (RO_2_–NO_2_), NO can either enhance autoxidation through the formation of reactive alkoxy radicals (RO) or terminate the radical chain *via* organic nitrate (RO–NO_2_) formation. In this work, we evaluate the binding affinities of NO_*x*_ toward RO_2_ molecules formed at different oxidation stages at DLPNO-CCSD(T)/aug-cc-pVTZ//ωB97X-D/aug-cc-pVTZ level of theory. This is followed by dissociation rate coefficient calculations using detailed balance and master equation simulations, indicating the lifetime and fragmentation pathways of the covalently bound RO_2_–NO_*x*_ molecules. The results suggest a tendency toward stronger RO_2_–NO_*x*_ stabilization with increasing oxidation stage, with a strong influence from molecular structure. This study also explores the rarely studied dissociation of RO_2_–NO_2_ into RO and NO_3_. Furthermore, by computing the binding enthalpies of NO_3_^−^ and 
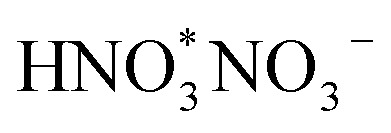
 with RO_2_–NO_*x*_, this work assesses the detectability of these N-containing oxidation products by the nitrate ion based chemical ionization mass spectrometer (NO_3_^−^-CIMS). The results are benchmarked against nitrophenol compounds known to form stable nitrate adducts. This analysis highlights potential detection biases associated with measuring nitrate-functionalized compounds using NO_3_^−^-CIMS as variations in molecular structure and clustering tendency influence signal intensities. Overall, these results highlight the regulatory roles of NO and NO_2_ in hydrocarbon oxidation and offer insights to improve interpretation of these oxygenated organic nitrates in atmospheric CIMS measurements.

## Abstract 2

Computational insights into NO and NO_2_ interactions with cyclohexene-derived RO_2_ radicals across different oxygenation stages, the resulting product stability, and NO_3_^−^-CIMS detection biases toward RO_2_–NO_*x*_ molecules.

## Graphical abstract

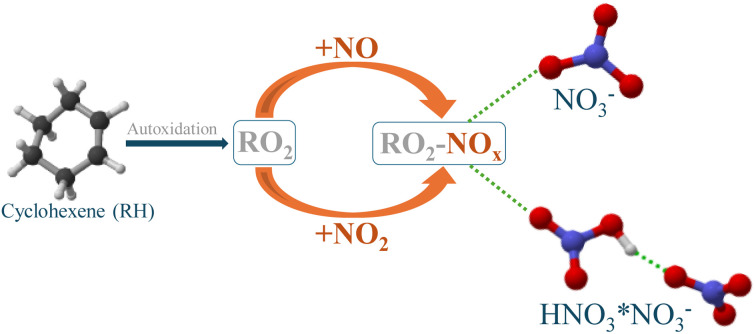

**Environmental significance**

Changing nitrogen oxide (NO_*x*_) levels worldwide are reshaping atmospheric oxidation chemistry. They alter the stability and reaction pathways of organic peroxy radicals (RO_2_), thereby influencing the formation of condensable organic products that contribute to secondary organic aerosol (SOA) formation. Cyclohexene, a commonly used surrogate for biogenic volatile organic compounds (BVOCs), forms RO_2_ radicals upon oxidation by ozone and OH, providing a representative model system for exploring RO_2_–NO_*x*_ chemistry. This theoretical study combines thermodynamic and kinetic analyses to elucidate RO_2_–NO_*x*_ molecular interactions and assess the detectability of their products using nitrate-ion chemical ionization mass spectrometry (NO_3_^−^-CIMS), supporting improved interpretation of atmospheric measurements.

## 1. Introduction

Biogenic volatile organic compounds (BVOCs) are the dominant source of non-methane hydrocarbon emissions to the atmosphere and significantly influence gas and heterogeneous-phase chemistry in the troposphere.^1,2^ Isoprene, monoterpenes, and related compounds are prominent examples of BVOCs.^3–5^ Due to their high reactivity and relatively short atmospheric lifetimes, typically ranging from minutes to hours, BVOCs undergo rapid chemical transformations through autoxidation processes. This leads to the formation of low-volatility oxygenated vapors known as highly oxygenated organic molecules (HOMs, common yields between 0.1% to 10%) which contribute to the formation of ambient secondary organic aerosol (SOA).^6,7^ These aerosols influence the distribution and abundance of atmospheric trace gases, affect the Earth's radiative balance, and thus play a role in climate regulation and atmospheric dynamics.^8,9^ Furthermore, SOA is the dominant fraction of atmospheric submicron aerosol linked to adverse health effects and resulting in increased mortality.^7,10–13^

**Fig. 1:**
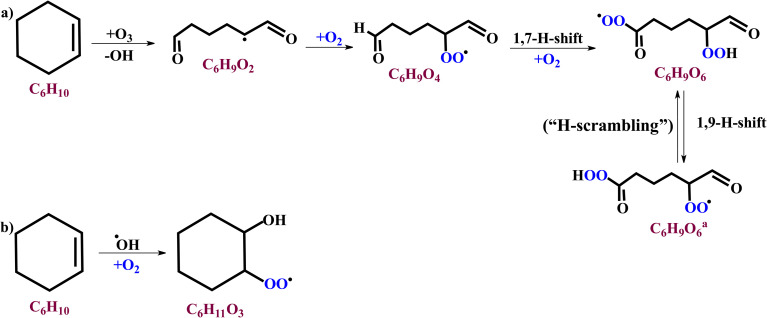
Reaction mechanisms for the formation of most abundant peroxy radicals from cyclohexene oxidation: (a) cyclohexene ozonolysis-initiated autoxidation, adapted from Rissanen *et al.*,^14^ leading to the formation of C_6_H_9_O_4_, C_6_H_9_O_6_, and C_6_H_9_O_6_^a^ as first generation peroxy radicals; and (b) the formation of the peroxy radical C_6_H_11_O_3_*via* OH addition to the double bond, followed by O_2_ addition.

Cyclohexene (C_6_H_10_) is widely utilized as a monoterpene surrogate due to its structural similarity, particularly its six-member carbon ring with an endocyclic double bond. Previously, Rissanen *et al.* reported an autoxidative reaction chain initiated by the reaction of C_6_H_10_ with ozone (O_3_), with around (4.5 ± 3.8)% HOM yield and occurring on a time scale of seconds using experimental and computational techniques.^14^ The mechanism begins with an O_3_ attacking the double bond of C_6_H_10_, forming a primary ozonide (POZ), which rapidly decomposes into one of two identical Criegee intermediates (CIs).^15–17^ They isomerize forming vinylhydroperoxides (VHPs) and subsequently dissociate releasing OH radicals. The formed carbon-centered alkyl radicals rapidly react with O_2_ yielding oxygen-centered peroxy radicals (RO_2_), C_6_H_9_O_4_ (Fig. 1a).^18,19^ These RO_2_ radicals can then undergo autoxidation, a sequence of successive unimolecular hydrogen shift reactions (H-shifts) followed by O_2_ additions, forming highly oxygenated peroxy radicals. When oxidation is terminated through unimolecular or bimolecular reactions, it leads to the formation of closed-shell highly oxygenated products.^20,21^ The co-produced OH radical from VHP decomposition can react with cyclohexene *via* both addition to the double bond and hydrogen abstraction pathways. At room temperature, the addition channel accounts for a larger fraction of the reaction (∼75%), while abstraction contributes up to ∼25%.^22^ In the present work, we focused on the OH addition pathway leading to the formation of the C_6_H_11_O_3_ peroxy radical following O_2_ addition (Fig. 1b).^23,24^

Among the generated primary RO_2_, a distinct acylperoxy radical (RC(O)OO) is also formed through a rapid H-shift from the aldehydic carbon atom. This radical exhibits significantly higher reactivity than typical RO_2_ radicals and undergoes exceptionally fast bimolecular reactions, making it a key player in atmospheric oxidation processes.^1,25–27^ The oxygenated peroxy radicals can recombine with other peroxy radicals (R′O_2_) present and form accretion products (ROOR′, so called “dimers”), with more carbon atoms than the parent VOC, yet not necessarily double.^6,25,28–30^ Additionally, RO_2_ can also react with hydroperoxy radicals (HO_2_), nitrogen oxides (NO_*x*_ = NO and NO_2_), and for example, OH radicals.^31,32^

When NO_*x*_ (NO and NO_2_) involvement is considered, they substantially perturb these oxidation sequences by altering RO_2_ lifetime and redirecting product distributions. This modulation is particularly important in polluted or urban-influenced environments, where NO_*x*_ levels are elevated and can shift SOA yields and oxidant budgets. At the molecular level, these effects arise from a set of characteristic RO_2_ + NO_*x*_ reactions. For example, Reaction (1) illustrates the general RO_2_ + NO reaction, forming a peroxy nitrite (RO_2_–NO) association product. This intermediate can either undergo rearrangement to form an organic nitrate (RO–NO_2_) (Reaction (2)), thereby terminating the oxidation sequence, or decompose into an alkoxy radical (RO) and NO_2_ (Reaction (3)), allowing the radical chain to propagate.^14,33–35^ Reaction (4) shows a common RO_2_ + NO_2_ reaction that terminates the oxidation chain by the formation of closed shell peroxynitrate (RO_2_–NO_2_). In the case of RC(O)OO reacting with NO_2_, the product is a peroxyacetyl nitrate (PAN), which is generally the most stable form of peroxynitrates, and the primary compound facilitating long-range transport of NO_*x*_.^25,35–37^

1 RO_2_ + NO → RO_2_–NO

2 RO_2_–NO → RO–NO_2_

3 RO_2_–NO → RO + NO_2_

4 RO_2_ + NO_2_ ⇌ RO_2_–NO_2_

The 2018 study by Rissanen highlighted the often-overlooked role of NO_2_ in atmospheric organic compound oxidation, emphasizing the possibility to suppress autoxidation sequences at different oxidation stages by reacting with oxygenated RO_2_.^25^ This interaction inhibits the formation of ROOR′ and can even outcompete the unimolecular rearrangements of acylperoxy radicals depending on NO_2_ conentrations.^14,21,24,38^ In contrast, NO exhibits a more complex influence on reaction pathways and it can accelerate the autoxidation process by generating alkoxy radicals (RO), which undergo hydrogen shifts (H-shifts) at significantly higher rates than RO_2_.^39^ These reactions must compete with other RO loss processes such as carbon chain fragmentation and hydrogen abstraction reaction by O_2_ forming closed-shell products.^27,40^ Experimental observations indicated that varying NO_2_ concentrations influence the mass spectrometric signals of oxidized product formation, highlighting the diverse roles of NO_*x*_ at different oxidation stages and leading to the formation of unique products. To fully understand these dependencies, a detailed analysis of the structural and thermodynamic properties of the molecular systems is essential. Therefore, we studied the interactions of NO and NO_2_ radicals with RO_2_ species formed from C_6_H_10_ oxidation at various oxidation stages. Among the various RO_2_ species formed during ozonolysis of C_6_H_10_ – including C_6_H_9_O_4_, C_6_H_9_O_6_, C_6_H_9_O_8_, and C_6_H_9_O_10_ – this study focuses on the earlier and more abundant radicals, C_6_H_9_O_4_ and C_6_H_9_O_6_ (Fig. 1a), as well as the isomer which forms *via* an extremely rapid 1,9-OOH H-shift (*i.e.*, hydrogen scrambling), predominantly converting hydroperoxy acyl peroxy radicals into peroxy peroxoic acid radicals.^41–43^ In OH initiated oxidation pathway, we focused on C_6_H_11_O_3_ (Fig. 1b) in line with the primary aim of probing RO_2_–NO_*x*_ interactions using quantum chemical calculations of reaction enthalpies (Δ*H*) and Gibbs free energies (Δ*G*). Subsequently, to evaluate the stability of the resulting RO_2_–NO_*x*_ molecules, we computed their dissociation rate constants into product channels.^44,45^

We also evaluated the detection sensitivity of nitrate chemical ionization mass spectrometry (NO_3_^−^-CIMS) for gas-phase products formed as peroxynitrates and organic nitrates (RO_2_–NO_2_ and RO–NO_2_) which includes C_6_H_11_O_2_NO_2_, C_6_H_11_O_3_NO_2_, C_6_H_9_O_3_NO_2_, C_6_H_9_O_4_NO_2_, C_6_H_9_O_5_NO_2_, C_6_H_9_O_6_NO_2_ C_6_H_9_O_5_^a^NO_2_, and C_6_H_9_O_6_^a^NO_2_. The likelihood of detecting these species depends on how strongly they bind to nitrate ions (NO_3_^−^) and nitric acid-nitrate reagent ion dimers (
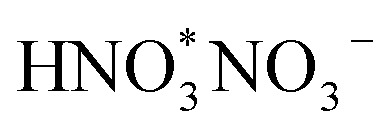
), relative to the binding affinity of HNO_3_ with these reagent ions.^45–48^ Molecules that form more stable clusters are more likely to be ionized and detected.^48,49^ Thus, to evaluate the detection characteristics of the target organic nitrates, we computed the binding enthalpies of these clusters and the corresponding nitrophenol clusters in order to provide a tangible comparison, and to allow a future follow-up study with authentic standard compounds. Notably, nitrophenol consistently appears as a dual peak in NO_3_^−^-CIMS spectra – one from direct NO_3_^−^ ionization and another from 
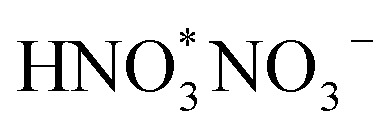
 clustering. If similar dual detection occurs for other nitrate-functionalized compounds, especially those with multiple nitrate groups, it may introduce quantification biases. These computational results provide valuable insight into potential detection artifacts in NO_3_^−^-CIMS, where compounds may be misidentified as having more nitrate groups than they contain, leading to uncertainties in understanding the role of NO_*x*_ in atmospheric oxidation chemistry.

## 2. Methods

Quantum chemical computations were utilized to determine the thermodynamic properties of RO_2_–NO_*x*_ molecules and their clusters with NO_3_^−^ and 
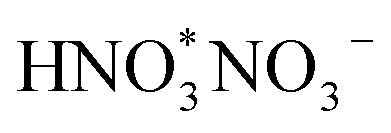
, additionally providing insight into their kinetic behavior. The calculations were initiated by finding the lowest energy conformers of the studied RO_2_, RO_2_–NO_*x*_ species, and their corresponding clusters with NO_3_^−^ and 
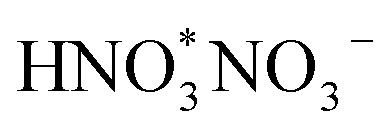
. Conformer sampling was performed in Spartan'24 using the Merck Molecular Force Field (MMFF) method, with torsions systematically varied across all rotatable bonds to ensure inclusion of all relevant conformers for further DFT refinement.^50^ To efficiently manage the large number of possible conformers in complex multicomponent systems, two different algorithms were used for conformer sampling. For the larger systems such as RO_2_–NO_*x*_ species clustered with NO_3_^−^ and 
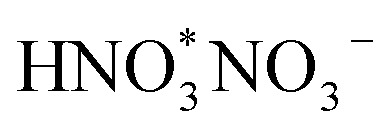
 – a MONTE CARLO algorithm was employed. For the smaller systems like RO, RO_2_, RO_2_–NO, RO–NO_2_, and RO_2_–NO_2_, a SYSTEMATIC search algorithm was used. After conformer sampling, single-point energy calculation was performed on all the conformers in Spartan at the B3LYP/6-31+G(d) level of theory.^51–53^ Conformers within 5 kcal mol^−1^ of the global minimum were retained, as higher-energy structures contribute negligibly to the Boltzmann distribution at 298 K. This cutoff is standard in conformational analyses, ensuring computational efficiency without loss of relevant thermodynamic information.^54,55^ These conformers were then optimized at the B3LYP/6-31+G(d) level of theory after which those within 2 kcal mol^−1^ of the global minimum were re-optimized and subjected to frequency calculations at the ωB97X-D/aug-cc-pVTZ level using Gaussian 16.^56,57^ To ensure that no low-energy conformers were missed, a metadynamics-based conformer search was performed using the GOAT workflow with the GFN2-xTB method and an energy cutoff of 6 kcal mol^−1^. For representative systems (C_6_H_11_O_3_NO, C_6_H_11_O_3_NO_2_, and C_6_H_11_O_2_NO_2_), the lowest-energy conformers obtained from GOAT and Spartan MMFF exhibit ZPE-corrected electronic energies differing by < 0.01 kcal mol^−1^ (Section S1, Table S1). Single-point electronic energy calculations were subsequently performed on the lowest-energy DFT geometries obtained from the MMFF conformer selection protocol using the domain-based local pair natural orbital coupled-cluster method (DLPNO-CCSD(T)), with the aug-cc-pVTZ basis set. TightPNO and TightSCF settings were employed to improve the description of electron correlation and weak dispersion interactions. The DLPNO calculations were performed with ORCA 5.0.3 and GOAT calculations with ORCA 6.0.1.^58,59^

The reliability of the single-reference treatment on these systems was assessed based on spin contamination, *T*_1_ and *D*_1_ diagnostics. The value of the total spin operator 〈*S*^2^〉 ranged from 0.750 to 0.751 for doublets, consistent with the expected ideal theoretical value of 0.75 indicating negligible spin contamination. In addition, for all closed-shell singlet intermediates we also performed unrestricted ωB97X-D/aug-cc-pVTZ calculations with guess = (mix,always) to verify that no hidden spin contamination is present. The resulting 〈*S*^2^〉 values (Section S1 Table S2) remained essentially 0.00, showing that both the restricted and unrestricted descriptions of the singlet species are free of spin contamination. The *T*_1_ diagnostic values calculated for the equilibrium geometries were found to be in the range of 0.020–0.024 for all studied species, including the RO_2_ and RO radicals, NO, NO_2_, and NO_3_, as well as the corresponding RO_2_–NO and RO_2_–NO_2_ molecules (see Section S1, Table S2). As *D*_1_ diagnostics cannot be evaluated within the DLPNO-CCSD(T)/aug-cc-pVTZ//ωB97X-D/aug-cc-pVTZ method, additional calculations were performed at the R(O)HF-RCCSD(T)-F12a/VDZ-F12//ωB97X-D/aug-cc-pVTZ level of theory for the open-shell systems. The resulting *T*_1_ values remained in the range of 0.020–0.024, close to the commonly used threshold of ∼0.02 for single-reference systems, while the *D*_1_ values ranged from 0.045 for the NO systems and 0.060 for the NO_2_ systems to 0.116–0.139 for NO_3_, RO and RO_2_ (Table S2). The elevated *D*_1_ values relative to the commonly used single-reference threshold of ∼0.05 indicate the presence of non-dynamical correlation arising from localized orbital mixing.^60^ In addition, *T*_1_ diagnostic values evaluated along the dissociation coordinate for representative systems (C_6_H_11_O_3_NO and C_6_H_11_O_3_NO_2_) were found to remain below 0.02 (Section S1, Table S3).^61,62^ Overall, the combined *T*_1_ and *D*_1_ diagnostics indicate the presence of some non-dynamical correlation effects, and therefore calculated results are interpreted qualitatively within a single-reference framework, while acknowledging the associated uncertainties due to possible multireference effects.^61,63,64^

The binding enthalpies (Δ*H*) and binding energies (Δ*E*) of NO and NO_2_ bonding with the peroxy radicals were calculated using eqn (5).

5 Δ*H*/Δ*E*_RO_2_−NO/NO_2__ = *H*/*E*_RO_2_−NO/NO_2__ − (*H*/*E*_RO_2__ + *H*/*E*_NO/NO_2__)

A comparison between ωB97X-D/aug-cc-pVTZ reaction enthalpies and DLPNO-CCSD(T)/aug-cc-pVTZ//ωB97X-D/aug-cc-pVTZ single-point-refined values evaluated on the DFT global minima is provided in Section S1, Table S4. DLPNO-CCSD(T)/aug-cc-pVTZ//ωB97X-D/aug-cc-pVTZ consistently predicts stronger binding enthalpies, with differences of approximately 0.9–5.3 kcal mol^−1^. These systematic shifts may reflect differences in the treatment of electron correlation and dispersion interactions between the methods, while preserving the relative trends across the studied systems. To further assess the reliability of the chosen level of theory, benchmark calculations using various methods were also performed on ethyl peroxy radical (C_2_H_5_O_2_) interacting with NO and NO_2_ as model systems. Binding energies (Δ*E*) and binding enthalpies (Δ*H*) were calculated using the general expression given in eqn (5). The reaction Gibbs free energy changes (Δ*G*) were evaluated for the corresponding intermediates (C_2_H_5_O_2_NO and C_2_H_5_O_2_NO_2_) using eqn (6) and (7), which describe their dissociation pathways.

6 Δ*G*_1_ = (*G*_RO_ + *G*_NO_2__) − *G*_RO_2_−NO_

7 Δ*G*_2_ = (*G*_RO_2__ + *G*_NO_2__) − *G*_RO_2_−NO_2__

The values obtained using the DLPNO-CCSD(T)/aug-cc-pVTZ//ωB97X-D/aug-cc-pVTZ approach were compared with R(O)HF-RCCSD(T)-F12a/VDZ-F12, canonical CCSD(T) calculations on ωB97X-D/aug-cc-pVTZ optimized geometries, as well as with DLPNO-CCSD(T)/aug-cc-pVTZ//M06-2X/aug-cc-pVTZ results to assess the effect of the underlying geometry (Section S1, Tables S5–S7). Relative to the canonical CCSD(T) and R(O)HF-RCCSD(T)-F12a reference calculations, DLPNO-CCSD(T)/aug-cc-pVTZ//ωB97X-D/aug-cc-pVTZ showed deviations of ∼0.7–2.1 kcal mol^−1^ for Δ*E* and Δ*H*, while Δ*G* deviations were within ∼0.2–3.9 kcal mol^−1^ for C_2_H_5_O_2_NO and ∼0.4–1.6 kcal mol^−1^ for C_2_H_5_O_2_NO_2_. With respect to DLPNO-CCSD(T)/aug-cc-pVTZ//M06-2X/aug-cc-pVTZ, the deviations were ≤0.05 kcal mol^−1^ for Δ*E* and Δ*H*, and ≤0.40 kcal mol^−1^ for the Δ*G* values of the possible dissociation channels, indicating only a minor dependence on the underlying optimized geometry. Overall, the observed deviations remain within a narrow range across the examined benchmark calculations, supporting the consistency of the computed energetics for the studied systems.^65^

Gibbs free energies for the systems studied in this work were calculated using both the rigid-rotor harmonic-oscillator (RRHO) approximation and a one-dimensional hindered-rotor (HR) treatment applied to the global minimum conformer at the ωB97X-D/aug-cc-pVTZ level using the Freq = HinderedRotor keyword.^66^ The resulting Gibbs free energies were subsequently corrected using DLPNO-CCSD(T)/aug-cc-pVTZ single-point electronic energies computed on the corresponding geometries.

To assess the molecular stabilities of association products, dissociation rate coefficients were computed. These rates were determined using the principle of detailed balance equating the ratio of the forward and backward reaction rates *via* equilibrium constant, as shown by eqn (8).^45^8
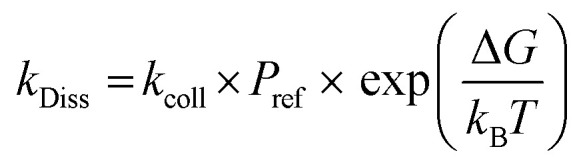
Here, *k*_Diss_ represents the dissociation (s^−1^) of the intermediates formed from the reaction of C_6_H_11_O_3_, C_6_H_9_O_4_, C_6_H_9_O_6_, and C_6_H_9_O_6_^a^ with NO_*x*_. Consistent with previous studies for radical–radical interactions collision-limited rate constant of *k*_coll_ = 2 × 10^−10^ cm^3^ molecule^−1^ s^−1^ was assumed in this study.^44,67,68^*P*_ref_ is the total concentration of molecules (*i.e.*, 2.46 × 10^19^ molecule cm^−3^), *k*_B_ is the Boltzmann constant, and *T* is the absolute temperature, 298.15 K. Δ*G* denotes the Gibbs free energy change for the association reaction for the corresponding dissociation channel, the free-energy change is equal in magnitude but opposite in sign.

To assess the collision-limit assumption used within the detailed-balance treatment for estimating the dissociation rate coefficients of the RO_2_–NO_*x*_ intermediates, relaxed scans were performed along the O(peroxy)-N(NO_*x*_) bond dissociation coordinate for representative C_6_H_11_O_3_NO and C_6_H_11_O_3_NO_2_ intermediates using unrestricted ωB97X-D and M06-2X methods with the aug-cc-pVTZ basis set and the keyword guess = (mix, always). Additional scans were also carried out using smaller basis set UωB97X-D/6-31+G* to assess the sensitivity of the results to the computational method. The relaxed scans showed monotonic increase in energy along the separation coordinate within the chemically relevant dissociation region (Section S1 Fig. S2). However, method-dependent differences were observed with increasing O–N separation accompanied by increasing spin contamination 〈*S*^2^〉 (after annihilation ∼0.0005–0.72), indicating increasing diradical character along the dissociation coordinate. We could not locate distinct maxima or minima corresponding to transition states or stabilized pre-reactive complexes using the present calculations. Consequently, the exact association rate coefficient could not be determined. A more rigorous characterization would require multireference or variational treatments, which are computationally demanding for the present C_6_ oxygenated RO_2_–NO_*x*_ systems. Therefore, following previous studies the RO_2_ + NO/NO_2_ association was treated within a collision-limit approximation (*k*_assoc_ ≈ *k*_coll_) for the qualitative kinetic analysis performed in this work.^44,68^ The sensitivity of this assumption was assessed by varying *k*_coll_ over the range 2 × 10^−10^ – 2 × 10^−12^ cm^3^ molecule^−1^ s^−1^.

In addition, considering the method-dependent variations observed in the DFT-level relaxed scans, further calculations were performed to assess the effect of the underlying DFT method on thermochemistry for selected C_6_ systems (C_6_H_11_O_3_NO/NO_2_ and C_6_H_9_O_4_NO/NO_2_). Noticeable differences were observed between ωB97X-D/aug-cc-pVTZ and M06-2X/aug-cc-pVTZ, particularly for the RO_2_–NO_2_ systems, where Δ*H* and Δ*G* differed by ∼3–5 kcal mol^−1^ (Section S1, Table S8). However, upon applying DLPNO-CCSD(T)/aug-cc-pVTZ single-point corrections to both geometries, these differences were reduced to within ∼0.7 kcal mol^−1^ for Δ*H* and ∼1.1 kcal mol^−1^ for Δ*G* (Section S1, Table S9).

We employed the master equation solver for multi-energy well reactions, MESMER (v7.1), to calculate dissociation rate coefficients of RO_2_–NO_*x*_ molecules.^69^ Association was implemented using the pre-exponential factor of 2 × 10^−10^ cm^3^ molecule^−1^ s^−1^ (*n* = 0, *E*_a_ = 0). To assess the sensitivity of the results, the pre-exponential factor was also varied over the range 2 × 10^−10^ – 2 × 10^−12^ cm^3^ molecule^−1^ s^−1^. An exponential-down energy transfer model was applied with an average energy transfer per collision of 〈Δ*E*_down_〉 = 225 cm^−1^ using N_2_ as the bath gas. Lennard-Jones parameters were set to *δ* = 6 and *ε* = 343 K. Simulations were carried out at 298.15 K and 760 Torr with an energy grain size of 50 cm^−1^. Partition functions were derived from ωB97X-D/aug-cc-pVTZ geometries. To enable direct comparison, low-frequency torsional modes were treated in two ways: within the harmonic oscillator (HO) approximation and as one-dimensional hindered rotors (HR). The HR treatment used Gaussian-calculated periodicities and barrier heights, specified the bonds corresponding to the hindered rotors, and removed the associated harmonic frequencies.

To assess the detection sensitivity of these molecules in NO_3_^−^-CIMS, we computed their binding enthalpies with NO_3_^−^ and 
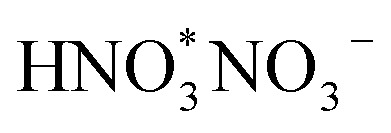
 which is a governing factor in their detectability with this technique. Molecules that bind more strongly to NO_3_^−^ than HNO_3_ can outcompete it and be detected with high sensitivity.^70^ Likewise, molecules with stronger binding to 
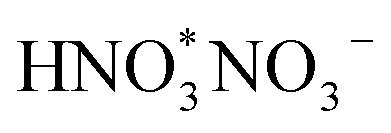
 than HNO_3_ itself are detectable *via* the dimer reagent ion. Δ*H* values were calculated using eqn (9) and (10) at the DLPNO-CCSD(T)/aug-cc-pVTZ//ωB97X-D/aug-cc-pVTZ level of theory. Note that the computed values represent cluster formation enthalpies, which are negative for favorable interactions. However, we report Δ*H* as positive values, defined as Δ*H* = |−Δ*H*|. Thus, higher (more positive) values correspond to stronger binding.

9 Δ*H*_binding_ = *H*_cluster with NO_3_^−^_ − (*H*_free molecules_ + *H*_NO_3_^−^_)

10
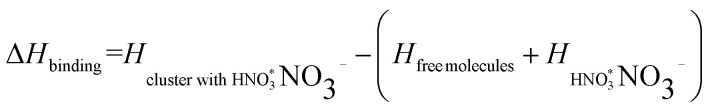


## 3. Results and discussions

### 3.1 Comparison of binding enthalpy: RO_2_–NO *vs.* RO_2_–NO_2_

**Fig. 2:**
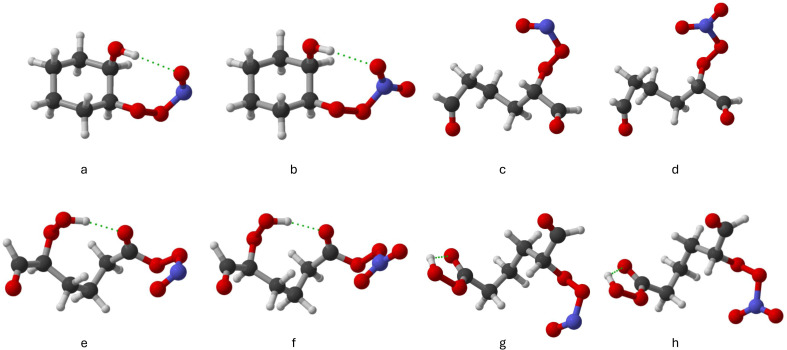
Optimized minimum energy geometries of the (a) C_6_H_11_O_3_NO, (b) C_6_H_11_O_3_NO_2_, (c) C_6_H_9_O_4_NO, (d) C_6_H_9_O_4_NO_2_, (e) C_6_H_9_O_6_NO, (f) C_6_H_9_O_6_NO_2,_ (g) C_6_H_9_O_6_^a^NO, and (h) C_6_H_9_O_6_^a^NO_2_ at ωB97X-D/aug-cc-pVTZ level of theory. Color coding: black is carbon, red is oxygen, blue is nitrogen, white is hydrogen, and green dashed line represents hydrogen bonding.

The relative binding strengths of RO_2_–NO_*x*_ intermediates play a crucial role in directing the course of the oxidation sequence. Small variations in binding strength can influence whether the reaction proceeds through autoxidation, or termination, thereby affecting the overall product distribution. Fig. 2a and b show the lowest-energy optimized geometries of the RO_2_–NO and RO_2_–NO_2_ product molecules, calculated at the ωB97X-D/aug-cc-pVTZ level of theory, formed through the OH addition pathway. Similarly, Fig. 2c–h present the lowest-energy conformers of molecules generated *via* the ozonolysis pathway, also optimized at the same level of theory.

The binding strengths of the peroxynitrites (Reaction (1)) and peroxynitrates (Reaction (4)) were assessed from their corresponding binding enthalpies (Table 1). For the RO_2_ species derived from C_6_H_11_O_3_, C_6_H_9_O_4_, C_6_H_9_O_6_, and C_6_H_9_O_6_^a^, the binding enthalpies with NO and NO_2_ radicals differ by 0.75 to 2.48 kcal mol^−1^ (C_6_H_11_O_3_: 0.98, C_6_H_9_O_4_: 0.75, C_6_H_9_O_6_: 2.48, C_6_H_9_O_6_^a^: 1.01).

**Table 1:** Comparison between the binding enthalpies (Δ*H* in kcal mol^−1^) of RO_2_–NO and RO_2_–NO_2_ molecules relative to RO_2_ + NO and RO_2_ + NO_2_ respectively

RO_2_	RO_2_–NO	RO_2_–NO_2_
	a Δ*H* (kcal mol^−1^)	a Δ*H* (kcal mol^−1^)
C_6_H_11_O_3_	25.68	24.71
C_6_H_9_O_4_	24.84	24.10
C_6_H_9_O_6_	33.34	30.86
C_6_H_9_O_6_^a^	26.07	25.06

a All the values were computed at DLPNO-CCSD(T)/aug-cc-pVTZ//ωB97X-D/aug-cc-pVTZ level of theory at 298.15 K including hindered-rotor corrections.

**Fig. 3:**
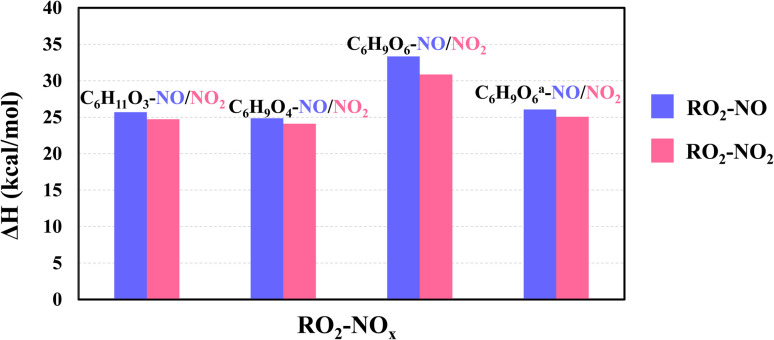
Comparison of binding enthalpies (Δ*H* kcal mol^−1^) for RO_2_-NO (blue) and RO_2_–NO_2_ (pink) molecules across different RO_2_ species.

This relatively narrow range demonstrates the competitive binding behavior of NO and NO_2_ toward RO_2_ radicals (Fig. 3). Greater oxygenation of RO_2_ species could typically be associated with stronger binding with NO_*x*_, owing to enhanced polarity and possible stabilizing interactions. However, by comparing C_6_H_11_O_3_ and C_6_H_9_O_4_, which represent primary oxidation products from the OH-initiated and ozonolysis pathways respectively, it is observed that C_6_H_11_O_3_ binds relatively more strongly to both NO and NO_2_, by 0.84 and 0.61 kcal mol^−1^ despite having one fewer oxygen atom. This enhanced binding can be rationalized by the presence of an OH substituent in C_6_H_11_O_3_, which provides additional stabilization likely through hydrogen bonding, as illustrated in Fig. 2a and b.

From O_4_ to O_6_, binding enthalpy increased, with acyl peroxy C_6_H_9_O_6_ showing stronger interactions than the secondary peroxy isomer (C_6_H_9_O_6_^a^ with peroxy acid group). For example, C_6_H_9_O_6_NO and C_6_H_9_O_6_NO_2_ bind more strongly than C_6_H_9_O_4_NO and C_6_H_9_O_4_NO_2_ by 8.49 and 6.76 kcal mol^−1^, respectively. In contrast, the secondary O_6_ peroxy radical showed modest increases in binding energy relative to C_6_H_9_O_4_ by 1.22 kcal mol^−1^ for NO and 0.96 kcal mol^−1^ for NO_2_. Thus, despite having the same number of oxygen atoms, the presence of an acyl peroxy group in C_6_H_9_O_6_ enhanced its binding by 7.27 kcal mol^−1^ with NO and 5.8 kcal mol^−1^ with NO_2_. Comparing C_6_H_9_O_6_ with C_6_H_11_O_3_ former binds stronger by 7.65 kcal mol^−1^ and 6.15 kcal mol^−1^ to NO and NO_2_, respectively while C_6_H_9_O_6_^a^ showed enhancement by 0.39 and 0.35 kcal mol^−1^ with NO and NO_2_ respectively. These findings highlight the greater stability of peroxyacyl nitrates (PANs), consistent with previous studies.^35,37^ All the RO_2_–NO_2_ exhibited comparable binding strengths to their RO_2_–NO analogues. The contrast between acyl and secondary peroxy radicals highlights the structural dependence of RO_2_–NO_*x*_ interactions. Acyl peroxy radicals, which contain a carbonyl group adjacent to the peroxy moiety, tend to form more strongly bound molecules with NO_*x*_ compared to secondary peroxy radicals. As a result, even low NO_*x*_ levels can efficiently stabilize or terminate the oxidation chain for acyl systems, whereas weaker-binding secondary peroxy radicals require higher NO_*x*_ concentrations to achieve comparable suppression. This structural sensitivity directly influences how NO_*x*_ modulates the oxidation sequence, ultimately determining product distributions at different oxidation stages, as observed in experimental studies.^25^

### 3.2 Dissociation fate of RO_2_–NO and RO_2_–NO_2_

The fate of RO_2_–NO_*x*_ molecules was studied by evaluating the Gibbs free energies of their dissociation pathway using eqn (6) and (7) as well as their rearrangement pathway to organic nitrates, RO–NO_2_. For RO_2_–NO, the relevant pathways include dissociation into RO + NO_2_, reversion to RO_2_ + NO, and rearrangement toward RO–NO_2_ formation. For RO_2_–NO_2_ , the dominant channel is generally reversion to reactants.^71^ In this work, we also investigated the potential fragmentation of RO_2_–NO_2_ into RO and NO_3_ (eqn (11)).

11 RO_2_–NO_2_ → RO + NO_3_

Decomposition of peroxyacyl nitrates has only been briefly addressed in previous work, whereas the fragmentation of non-acyl RO_2_–NO_2_ into RO and NO_3_ has, to the best of our knowledge, received no prior attention.^72^ Here, Table 2 presents the calculated reaction Gibbs free energies for all the possible fates of RO_2_–NO and RO_2_–NO_2_ molecules. The calculated Δ*G* values provide insight into the relative thermodynamic feasibility of their dissociation, reversion, and rearrangement channels as peroxy radicals become more oxidized. The Δ*G* values presented here include hindered-rotor (HR) corrections. A comparison with values from the pure harmonic oscillator (HO) treatment is provided in the Section S1, Tables S10 and S11. In Table 2 the superscripts I and II in Δ*G* denote constrained and unconstrained optimizations, respectively, and are discussed in detail in the subsequent section.

**Table 2:** Reaction Gibbs free energies (Δ*G* kcal mol^−1^) for the reversion, dissociation, and isomerization pathways of RO_2_–NO and RO_2_–NO_2_

RO_2_	RO_2_–NO			RO_2_–NO_2_	
	aΔ*G* for RO_2_ + NO (kcal mol^−1^)	aΔ*G* for RO + NO_2_ (kcal mol^−1^)	aΔ*G* for RO–NO_2_ (kcal mol^−1^)	aΔ*G* for RO_2_ + NO_2_ (kcal mol^−1^)	aΔ*G* for RO + NO_3_ (kcal mol^−1^)
C_6_H_11_O_3_	13.84	2.09	−25.95	11.07	26.81
C_6_H_9_O_4_	13.11	1.71	−25.97	10.79	26.88
C_6_H_9_O_6_	21.82	−6.51^I^	−20.61	17.53	15.11^I^
		−31.43^II^			−9.80^II^
C_6_H_9_O_6_^a^	14.49	2.67	−24.29	11.59	27.26

a All values were obtained at the DLPNO-CCSD(T)/aug-cc-pVTZ//ωB97X-D/aug-cc-pVTZ level of theory at 298.15 K, including one-dimensional hindered-rotor corrections. ^I^Δ*G* values correspond to constrained optimizations performed at the ωB97X-D/aug-cc-pVTZ level, where a C–C bond is constrained that prevents the direct elimination of CO_2_ during RO optimization, thereby leading to RO + NO_2_ and RO + NO_3_, respectively. ^II^Δ*G* values correspond to the unconstrained case, allowing spontaneous CO_2_ loss, computed at the ωB97X-D/aug-cc-pVTZ level. In this pathway, RO_2_-NO dissociates R + CO_2_ + NO_2_, while RO_2_–NO_2_ dissociates ultimately R + CO_2_ + NO_3_.

In line with previous studies, once RO_2_–NO is formed, it is thermodynamically unstable with respect to dissociation into RO + NO_2_, which is the most favorable channel across all studied peroxy radicals (Δ*G* ∼1.7–2.7 kcal mol^−1^), whereas decomposition back to RO_2_ + NO is much less favorable (Δ*G* >13 kcal mol^−1^).^34,71,73,74^ The rearrangement toward RO–NO_2_ is very exergonic (Δ*G* between −26 to −20 kcal mol^−1^), showing that the nitrate channel represents a deep thermodynamic sink along all the studied RO_2_.^73,75^ For RO_2_–NO_2_, reversion back to RO_2_ + NO_2_ has Δ*G* values of about 11 kcal mol^−1^, whereas fragmentation to RO + NO_3_ is thermodynamically highly unfavourable with Δ*G* around 22–23 kcal mol^−1^. With increasing oxidation stage, the thermodynamic driving force for RO_2_–NO_2_ dissociation became less favorable.

In Table 2, the subscripted values (I and II) correspond to two treatments of CO_2_ elimination from acylperoxy-NO_*x*_ dissociation channel. Case (I) refers to constrained optimizations at the ωB97X-D/aug-cc-pVTZ level, where CO_2_ loss was artificially prevented, such that dissociation proceeds simply as RO + NO_2_ or RO + NO_3_. These constrained results provide a reference that allows direct comparison with non-acyl peroxy radicals (Section S1, Table S12). Case (II) corresponds to the unconstrained optimizations at the ωB97X-D/aug-cc-pVTZ level, where CO_2_ elimination occurs spontaneously during optimization, leading to reaction (12) and (13).

12 RO_2_–NO → R(C_5_H_9_O_3_) + NO_2_ + CO_2_

13 RO_2_–NO_2_ → R(C_5_H_9_O_3_) + NO_3_ + CO_2_

As Δ*G* is a state function, both constrained and unconstrained values are meaningful and provide complementary views of the underlying thermodynamics. In the unconstrained optimizations, CO_2_ elimination appears as a highly exergonic process, suggesting a much deeper thermodynamic sink. However, this behaviour may also reflect the inability of single-reference DFT methods to properly stabilize the RO intermediates. Since the actual barrier for the R–CO_2_ bond cleavage has not been determined, the atmospheric relevance of this channel remains uncertain based on the present data alone.

Using Δ*G* within the detailed balance framework (eqn (8)), we calculated dissociation rate constants based on a collision-limit association rate of *k*_coll_ = 2 × 10^−10^ cm^3^ molecule^−1^ s^−1^ (Table 3), and the results presented here are based on this value. To complement these upper-bound estimates, dissociation rate constants were also evaluated with MESMER. To assess the sensitivity of the kinetic parameters, *k*_coll_ was varied over the range 2 × 10^−10^ – 2 × 10^−12^ cm^3^ molecule^−1^ s^−1^ in the detailed-balance framework, and the pre-exponential factor in MESMER was varied over an equivalent range. While these variations affect the absolute rate constants, the relative trends between reaction channels and the overall chemical behaviour remained unchanged (see Section S1, Tables S13 and S14).

**Table 3:** Dissociation rates (*k*_Diss_) of RO_2_–NO in s^^−^1^

RO_2_–NO	RO_2_–NO → RO_2_ –NO			RO_2_–NO → RO + NO_2_		
	b *k* ^Detailed balance^_diss_	c *k* ^MESMER-HO^_diss_	d *k* ^MESMER-HR^_diss_	b *k* ^Detailed balance^_diss_	c *k* ^MESMER-HO^_diss_	d *k* ^MESMER-HR^_diss_
C_6_H_11_O_3_NO	3.48 × 10^−01^	2.02 × 10^−01^	3.55 × 10^−02^	1.43 × 10^08^	1.26 × 10^06^	9.56 × 10^05^
C_6_H_9_O_4_NO	1.20 × 10^00^	3.66 × 10^−01^	1.42 × 10^−02^	2.72 × 10^08^	3.66 × 10^06^	1.62 × 10^06^
C_6_H_9_O_6_NO	4.95 × 10^−07^	1.11 × 10^−07^	—	—	—	—
C_6_H_9_O_6_^a^NO	1.17 × 10^−01^	1.95 × 10^−02^	—	5.40 × 10^07^	1.99 × 10^06^	—

b Detailed balance with Δ*G* obtained at the DLPNO-CCSD(T)/aug-cc-pVTZ//ωB97X-D/aug-cc-pVTZ level of theory including hindered-rotor (HR) corrections.

c MESMER assuming harmonic oscillators (*k*^MESMER-HO^_diss_),

d MESMER with HR treatment (*k*^MESMER-HR^_diss_). All values are reported at 298.15 K using a representative collision rate of 2 × 10^–10^ cm^3^ molecule^−1^ s^−1^.

As summarized in Table 3, the MESMER-derived rates for RO_2_–NO → RO + NO_2_ are systematically lower than the corresponding detailed-balance estimates, although the reaction remains rapid in all cases. The detailed-balance approach yields the highest rate constants, while MESMER calculations using the harmonic-oscillator (HO) approximation give consistently lower values. Incorporating explicit hindered-rotor (HR) corrections further decreases the rates by roughly an order of magnitude. The MESMER-HR rates remain on the order of 10^5^–10^6^ s^−1^, implying that once formed, RO_2_–NO molecules are inherently short-lived under atmospheric conditions.

Since the rearrangement of RO_2_–NO to RO–NO_2_ is exergonic (Table 2) and organic nitrates were observed experimentally, and the RO_2_ + NO back-dissociation pathways are further predicted to be negligible based on their calculated dissociation rate coefficients, the fate of the transient RO_2_–NO intermediates are expected to be governed primarily by competition between RO + NO_2_ formation and rearrangement to RO–NO_2_.^76^ Assessing the relative importance of these pathways requires kinetic information on the rearrangement barrier height. Previous studies have shown that the rearrangement pathway becomes increasingly competitive with dissociation as the size of the R group increases, making the rearrangement channel non-negligible for substituents containing at least two carbon atoms.^77,78^ Accordingly, to assess the relative importance of the competing RO + NO_2_ and RO–NO_2_ formation channels, approximate branching ratios were estimated using nitrate yields obtained from the Jenkin *et al.* parameterization.^71^ The estimated nitrate yield for C_6_H_11_O_3_NO (∼14.06%) falls within the experimental range (21 ± 8%) reported by Teng *et al.* for C_6_ β-hydroxy peroxy radical systems, providing an experimental reference point for comparison.^71,76^ Lower estimated yields (∼7–8%) were obtained for C_6_H_9_O_4_NO and C_6_H_9_O_6_^a^NO and are consistent with the structure-dependent behaviour observed experimentally for related C_6_ systems (internal alkenes).^76^ Within the applied parameterization, nitrate formation from C_6_H_9_O_6_NO intermediate was predicted to be negligible and was therefore not considered. A possible explanation could be rapid CO_2_ elimination from the transient acyloxy radical, competing with completion of the nitrate-forming rearrangement pathway. However, obtaining accurate quantitative branching ratios would require rigorous characterization of the nitrate-forming pathway by calculating its associated kinetics using multireference approach which is beyond the scope of the present work.

In the case of RO_2_–NO_2_ (non-PAN species) (Table 4), dissociation back to RO_2_ + NO_2_ is consistently faster than fragmentation to RO + NO_3_ which has a rate of ≤10^−10^ s^−1^ with all computational approaches and is therefore non-competitive. Based on Δ*G*-derived detailed balance, the reverse rate increases from the O_3_ to the O_4_ species, and then drops for the O_6_ species where C_6_H_9_O_6_NO_2_ shows a strongly reduced rate of decomposition, consistent with its PAN-like character noted in a previous study.^25^

**Table 4:** Dissociation rates (*k*_Diss_) of RO_2_–NO_2_ in s^−1^

RO_2_–NO_2_	RO_2_–NO_2_ → RO_2_ + NO_2_			RO_2_–NO_2_ → RO + NO_3_		
	b *k* ^Detailed balance^_diss_	*ck* ^MESMER-HO^_diss_	d *k* ^MESMER-HR^_diss_	*bk* ^Detailed balance^_diss_	*ck* ^MESMER-HO^_diss_	d *k* ^MESMER-HR^_diss_
C_6_H_11_O_3_NO_2_	3.73 × 10^01^	1.66 × 10^01^	1.01 × 10^01^	1.07 × 10^−10^	6.09 × 10^−11^	3.51 × 10^−11^
C_6_H_9_O_4_ NO_2_	6.03 × 10^01^	1.59 × 10^01^	3.31 × 10^−01^	9.66 × 10^−11^	2.93 × 10^−11^	5.98 × 10^−13^
C_6_H_9_O_6_ NO_2_	6.92 × 10^−04^	1.21 × 10^−04^	—	—	—	—
C_6_H_9_O_6_^a^ NO_2_	1.54 × 10^01^	2.16 × 10^00^	3.49 × 10^−02^	5.04 × 10^−11^	8.33 × 10^−12^	1.34 × 10^−13^

b Detailed balance with Δ*G* obtained at the DLPNO-CCSD(T)/aug-cc-pVTZ//ωB97X-D/aug-cc-pVTZ level of theory including hindered-rotor (HR) corrections.

c MESMER assuming harmonic oscillators (*k*^MESMER-HO^_diss_),

d MESMER with HR treatment (*k*^MESMER-HR^_diss_). All values are reported at 298.15 K using a representative collision rate of 2 × 10^−10^ cm^3^ molecule^−1^ s^−1^.

MESMER-HO calculations reproduce the same overall sequence, and the absolute differences between O_3_ and O_4_ remain modest, indicating broadly comparable dissociation rates at this level of treatment. While applying HR corrections further lowers the dissociation rates consistent with reduced torsional entropy, but the effect is unusually strong for C_6_H_9_O_4_NO_2_. Under MESMER-HR, O_4_ is predicted to dissociate more slowly than O_3_, which appears opposite to what might be expected from their relative thermodynamics. This difference may be explained by the O_4_ system having more low-frequency torsional modes (8 hindered rotors) compared to O_3_ (3 hindered rotors), so the hindered-rotor treatment introduces a larger entropic penalty in O_4_.

For the O_6_ species, the HR treatment could only be applied reliably to C_6_H_9_O_6_^a^NO_2_, while for C_6_H_9_O_6_NO, C_6_H_9_O_6_^a^NO, and C_6_H_9_O_6_NO_2_, the Freq = HinderedRotor analysis failed to identify the torsional modes. To enable an approximate HR correction, we transferred the torsional barrier heights and periodicities from C_6_H_9_O_6_^a^NO_2_ by analogy. This estimated dissociation rates of 2.4 × 10^−10^ s^−1^ for C_6_H_9_O_6_NO, 5.5 × 10^−4^ s^−1^ for C_6_H_9_O_6_^a^NO (RO_2_–NO → RO_2_ + NO), and 9.1 × 10^−8^ s^−1^ for C_6_H_9_O_6_NO_2_ (RO_2_–NO_2_ → RO_2_ + NO_2_).

In the case of acyl peroxy radicals such as C_6_H_9_O_6_NO and C_6_H_9_O_6_NO_2_ dissociation *via* pathways that generate alkoxy radicals lead to rapid CO_2_ elimination (reaction (12) and (13)), as indicated by DFT calculations. Using constrained optimizations at the ωB97X-D/aug-cc-pVTZ level, the Δ*G*-based (Table 2(^I^)) dissociation rates for C_6_H_9_O_6_NO_2_ were 3.95 × 10^−2^ s^−1^ for C_6_H_9_O_6_NO_2_ → RO + NO_3_ (Section S1, Table S15). At this level of theory, the dissociation of acyl peroxy nitrates appears comparatively fast. However, studies of other RO_2_–NO_2_ species at the same DFT level have shown that dissociation rates can be substantially reduced when refined using DLPNO-CCSD(T) corrections and MESMER with hindered-rotor treatment. A similar behavior may therefore apply to the present acyl peroxy nitrate system. Nevertheless, due to rapid CO_2_ loss following alkoxy formation after NO_2_ and NO_3_ elimination from C_6_H_9_O_6_NO and C_6_H_9_O_6_NO_2_, accurately determining these rates remains challenging. These uncertainties also motivate future investigation into whether PAN-like (C_6_H_9_O_6_NO_2_) species can contribute to minor NO_3_ radical formation under certain structural or energetic conditions.

**Fig. 4:**
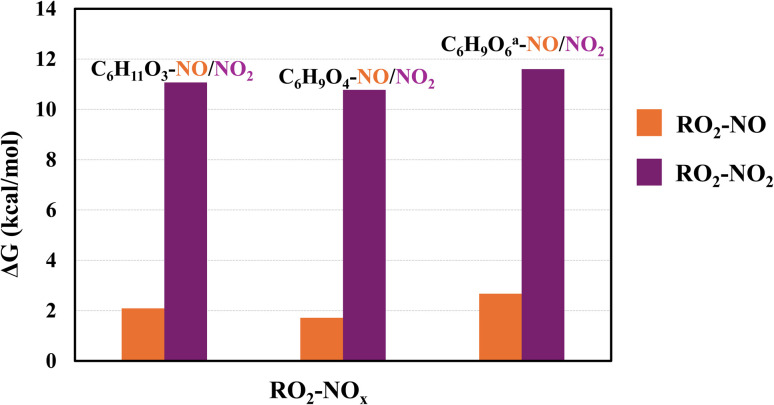
Reaction Gibbs free energy for dissociation of non-PAN species, RO_2_–NO (brick-red) and RO_2_–NO_2_ (dark purple) leading to their dominant dissociation channels (RO_2_–NO → RO + NO_2_; RO_2_–NO_2_ → RO_2_ + NO_2_).

Thermodynamic and kinetic analyses indicate that RO_2_–NO_2_ can persist relatively longer than RO_2_–NO in the reaction sequence, thereby functioning as temporary NO_*x*_ reservoirs. Fig. 4 illustrates this trend by showing the Gibbs free energy variation along the dissociation pathways for non-PAN molecules, where the NO_2_-loss channels are more stabilized than the corresponding NO-loss pathways. Consequently, the RO_2_–NO_2_ adducts may act as temporary NO_*x*_ reservoirs in the atmosphere.

This behavior may extend to more highly oxidized and functionalized peroxy radicals (*e.g.*, C_6_H_9_O_8_ and C_6_H_9_O_10_) which may exhibit increased binding enthalpy and stability toward NO_2_ depending on their molecular structure. These computational insights are consistent with atmospheric chamber studies, which have demonstrated that a significant fraction of non-acyl RO_2_–NO_2_ can persist under atmospherically relevant conditions, with enhanced stability and accumulation observed at lower temperatures.^79^ These observations, together with our computed values, emphasize the importance of NO_2_ chemistry alongside NO for both acyl and non-acyl peroxy radicals. Incorporating the reactivity of RO_2_–NO_2_ intermediates in the atmospheric models that primarily focus on NO-species or PANs will provide a more comprehensive view of oxidation processes in the atmosphere.

### 3.3 Modelling the detection of peroxy-NO_*x*_ molecules using nitrate chemical ionization mass spectrometry

Autoxidation-produced polyperoxide HOMs are detected sensitively using NO_3_^−^-CIMS; however, only a limited number of species with lower oxygen content have been observed.^14,46^ This lack of detection is attributed to weaker binding affinities of the less oxidized products to the reagent ions. To allow a future experimental assessment of the detection sensitivities of the targeted cyclohexene derived RO/RO_2_–NO_2_ oxidation products, we conducted a comparative analysis of binding enthalpies of a set of standard compounds containing 2-nitrophenol (2-NP), 3-nitrophenol (3-NP), 4-nitrophenol (4-NP) and HNO_3_ with NO_3_^−^ and 
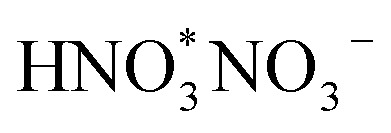
 reagent ions. The inclusion of nitrophenol derivatives as model compounds is partly due to their persistent observations during ambient field deployments and partly due to the presence of the NO_2_ functional group, which is expected to exhibit similar reactivity in ion–molecule interactions as the RO/RO_2_–NO_2_ species considered in this study.^80^

A detailed comparison of the binding enthalpies of clusters formed by RO–NO_2_ and RO_2_–NO_2_ (products from Reactions (2) and (4)) with NO_3_^−^ and 
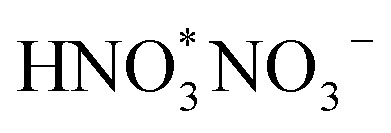
, along with various nitrophenol derivatives and nitric acid, is presented in Table 5. The binding enthalpies obtained for the standard compounds 4-NP and HNO_3_ with NO_3_^−^, align closely with the values reported by Hyttinen *et al.* with minor differences attributable to the differences in used basis sets.^46^ The binding capacity of 3-NP towards NO_3_^−^ was found to be lower than that of 4-NP but higher than that of HNO_3_. In contrast, 2-NP exhibits significantly lower binding enthalpy, with reductions of 9.20 kcal mol^−1^, 8.76 kcal mol^−1^, and 8.03 kcal mol^−1^ relative to 4-NP, 3-NP, and HNO_3_, respectively. This substantial decrease is attributed to the well-known strong internal hydrogen bonding in 2-NP, which limits its ability to interact effectively with NO_3_^−^ and results in poor experimental detection sensitivity. A similar trend of the weakest interaction is observed when 2-NP forms cluster with 
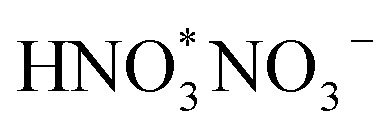
.

**Table 5:** Binding enthalpies (Δ*H*) of RO/RO_2_–NO_2_ molecules, nitrophenols (2-NP, 3-NP, 4-NP), and HNO_3_ with NO_3_^−^ and 
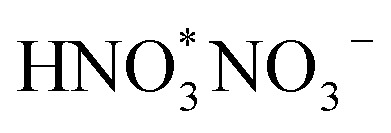
 in kcal mol^−1^

Systems	aΔ*H* (kcal mol^−1^)	aΔ*H* (kcal mol^−1^)
	System-NO_3_^−^	System- 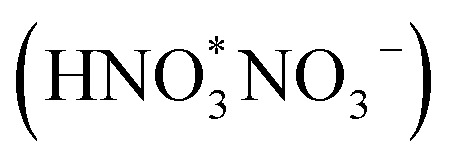
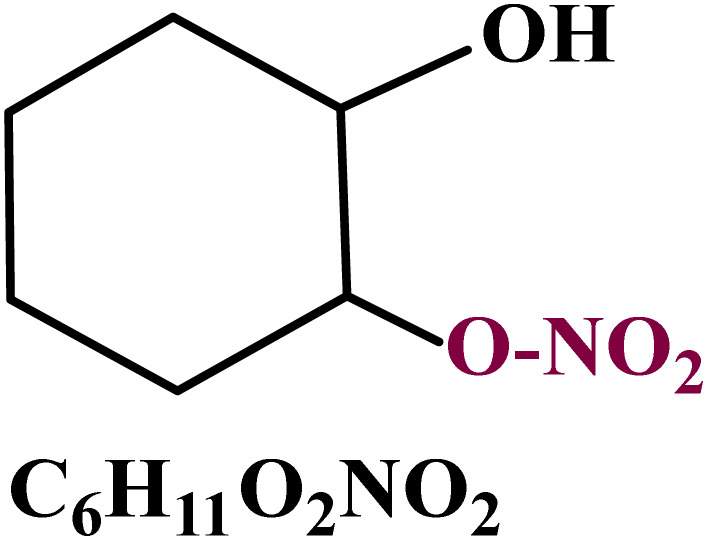	22.32	15.53
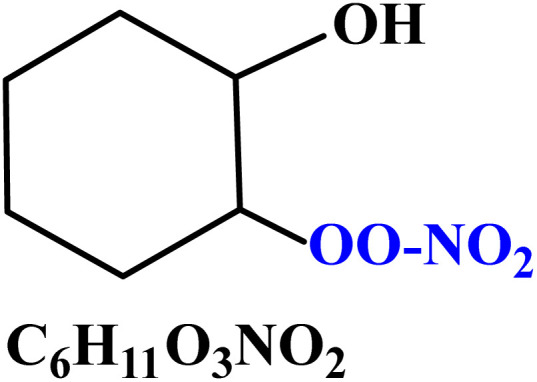	21.11	13.92
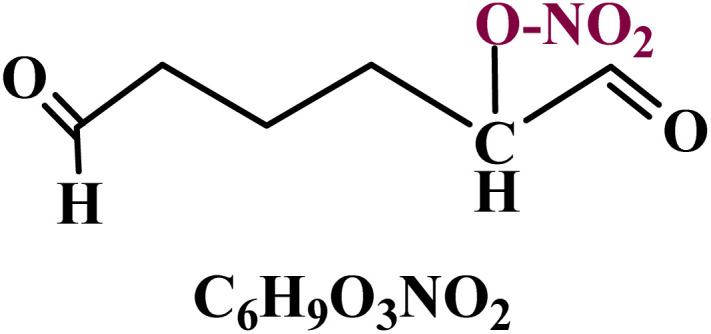	26.49	21.41
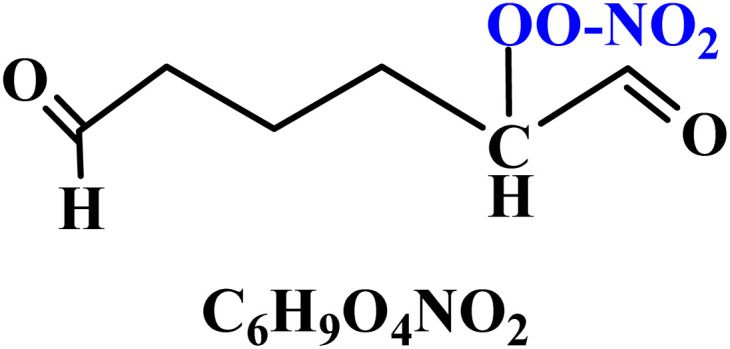	26.58	21.58
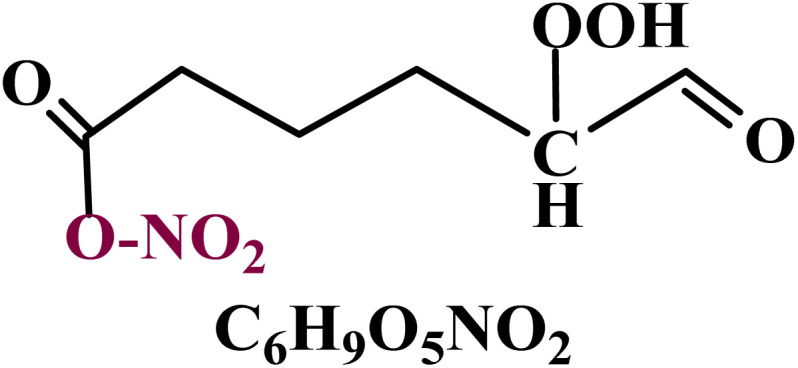	30.08	23.07
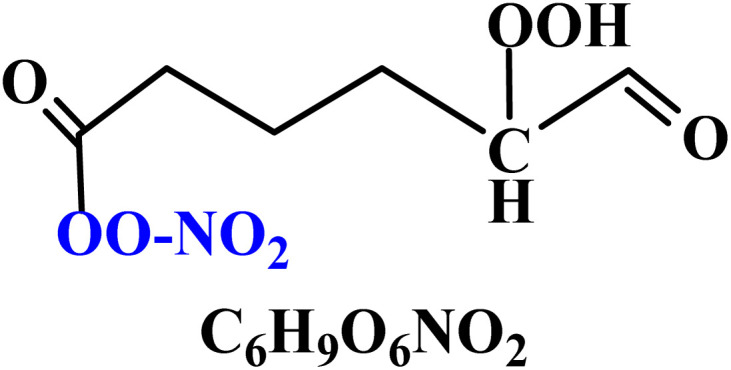	30.14	22.85
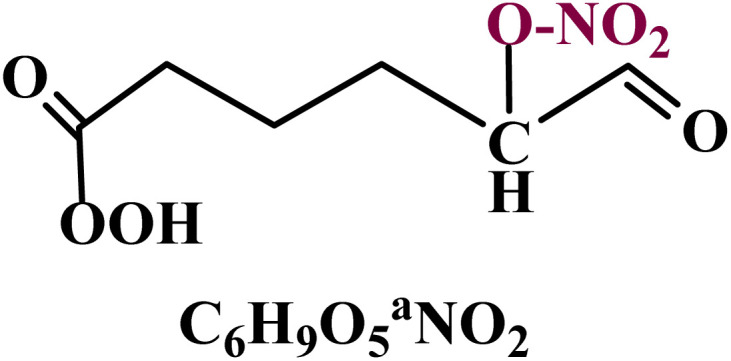	34.27	25.67
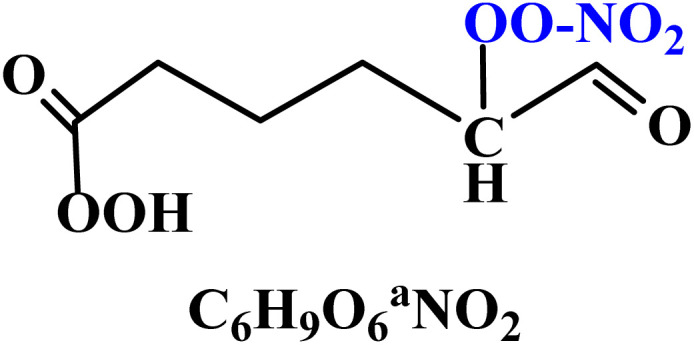	32.50	24.38
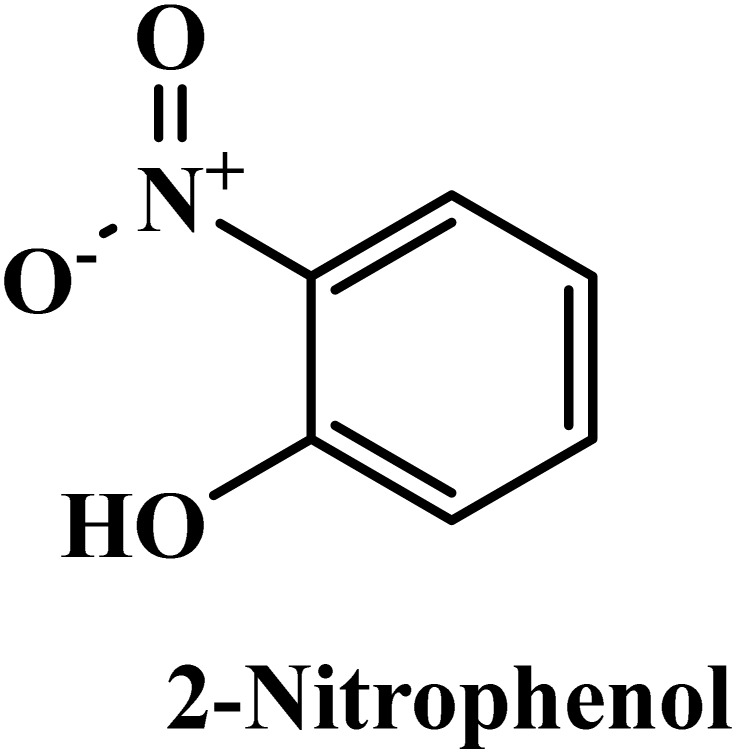	21.20	12.37
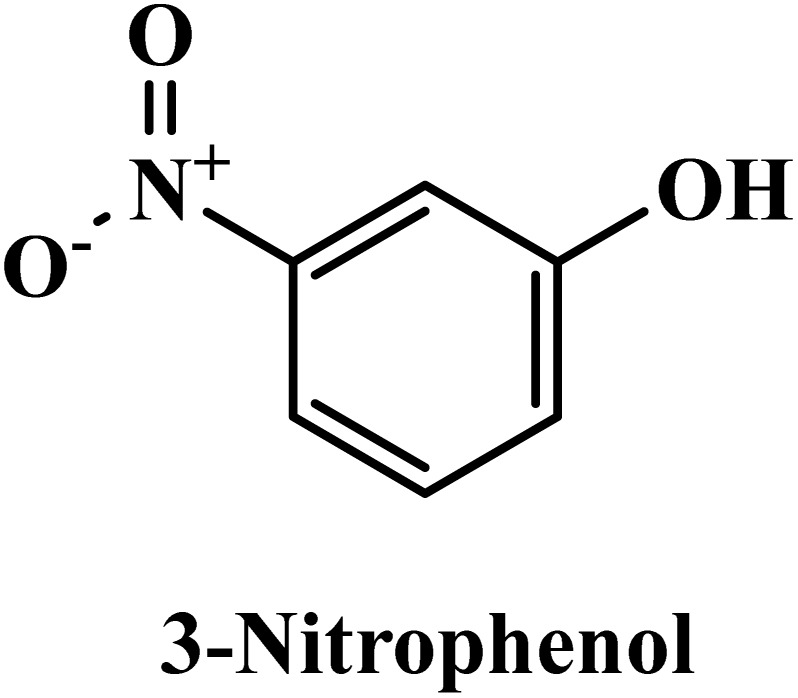	29.96	22.61
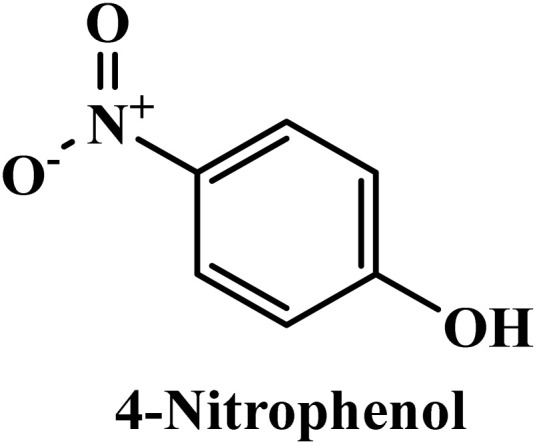	30.44 (31.07)^I^	22.14
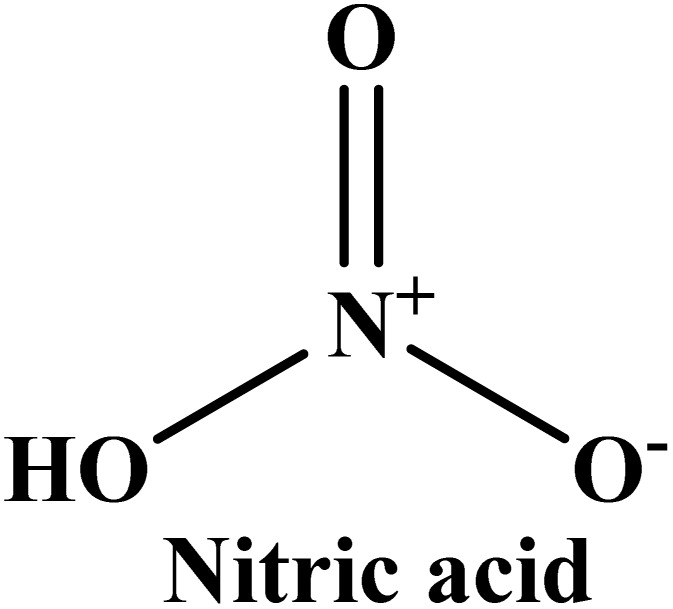	29.83 (30.91)^II^	19.83

a All values are calculated at the DLPNO-CCSD(T)/aug-cc-pVTZ//ωB97X-D/aug-cc-pVTZ level of theory at 298.15 K. ^I^Binding enthalpy for 4-nitrophenol and ^II^binding enthalpy for nitric acid calculated at the DLPNO-CCSD(T)/def2-QZVPP//ωB97X-D/aug-cc-pVTZ level of theory, as reported by Hyttinen *et al.*^46^

The computed clustering energies of early generated RO-NO_2_ species C_6_H_11_O_2_NO_2_, C_6_H_11_O_3_NO_2_, C_6_H_9_O_3_NO_2_, and C_6_H_9_O_4_NO_2_ with NO_3_^−^ are 3.25 to 8.72 kcal mol^−1^ lower than that of HNO_3_. Similarly, when compared to the nitrophenol references their clustering enthalpies are also 3.38 to 8.85 kcal mol^−1^ and 3.86 to 9.33 kcal mol^−1^ lower than those of 3-NP and 4-NP, respectively. These results imply a low experimental detection sensitivity of NO_3_^−^ for less oxygenated RO–NO_2_. The latter generated C_6_H_9_O_5_NO_2_, C_6_H_9_O_5_^a^NO_2_, C_6_H_9_O_6_NO_2_ and C_6_H_9_O_6_^a^NO_2_ nitrates which all contain an additional hydroperoxide group, however, exhibit enhanced clustering enthalpies with NO_3_^−^ ranging from 0.22 to 4.44 kcal mol^−1^ above that of HNO_3_, and consequently imply a high detection sensitivity. When benchmarked against 3-NP they exhibit 0.12 to 4.31 kcal mol^−1^ stronger binding with NO_3_^−^ and relative to 4-NP the enhancement ranges from 1.64 to 3.83 kcal mol^−1^. Despite sharing the same molecular formulae, these differences in binding enthalpy can be attributed to variations in molecular structure, which influence both the strength and geometry of interaction with the reagent ion in CIMS.

To understand the significance of the dual peaks observed in several NO_3_^−^-CIMS experiments, where total product signal intensity arises from a combination of ions formed *via* clustering with the reagent ion monomer and dimer, we also evaluated the binding enthalpies of the target molecules with the 
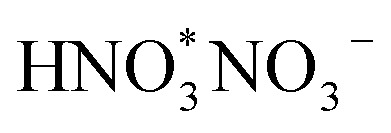
 reagent ion dimer. Thus, these values help assess the likelihood of cluster formation with 
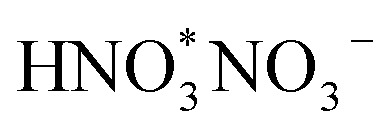
 which could lead to an additional peak in the mass spectrum. Interestingly, all except the least oxygenated nitrates show a higher binding enthalpy with 
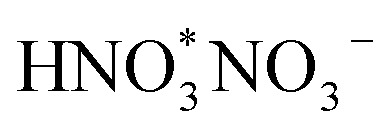
 than HNO_3_, illustrating the potential for misidentification. The less oxygenated species, C_6_H_11_O_2_NO_2_ and C_6_H_11_O_3_NO_2,_ show significantly weaker binding to 
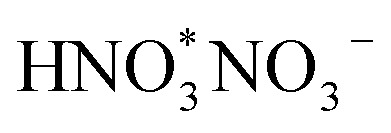
 with clustering enthalpies from 4.33 to 8.69 kcal mol^−1^ lower than those of HNO_3,_ 3-NP and 4-NP suggesting they are unlikely to be detected *via* the reagent ion dimer. In contrast, C_6_H_9_O_3_NO_2_ and C_6_H_9_O_4_NO_2_ exhibited moderate binding enhancements by 1.58 to 1.75 kcal mol^−1^ greater than HNO_3_, but lower than 3-NP and 4-NP by 0.56 to 1.2 kcal mol^−1^. Despite this, their stronger binding compared to HNO_3_ indicates a high likelihood of detection *via* the 
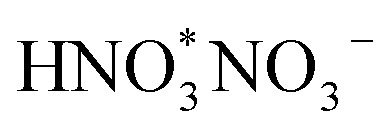
 reagent dimer. This trend becomes even more pronounced for the higher oxygenated compounds C_6_H_9_O_5_NO_2_, C_6_H_9_O_5_^a^NO_2,_ C_6_H_9_O_6_NO_2_ and C_6_H_9_O_6_^a^NO_2_, where the binding enthalpies exceed those of all reference standards (3-nitrophenol, 4-nitrophenol, and HNO_3_) by 0.24 to 5.84 kcal mol^−1^. It should also be emphasized that according to the current results the secondary RO–NO_2_ formed from the 6 O-atom containing peroxy radicals bind significantly stronger to the reagent ion dimer than the analogous acyl-RONO_2_ (*i.e.*, 1.53 and 2.60 kcal mol^−1^), indicating their higher tendency to participate in subsequent cluster growth.

**Fig. 5:**
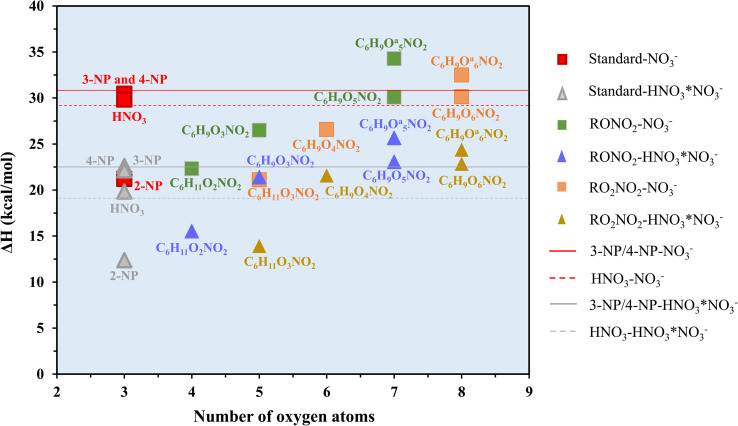
The comparison of binding enthalpies of RO–NO_2_ and RO_2_–NO_2_ with NO_3_^−^ and 
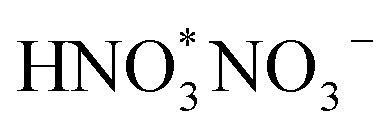
 are plotted against number of oxygen atoms, alongside standards with three oxygen atoms. Squares represent clustering with NO_3_^−^: red for standards, green for RO–NO_2_ and orange for RO_2_–NO_2_. Triangles represent 
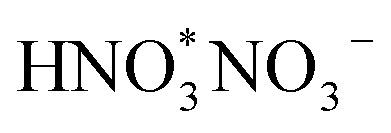
 clustering: grey for standards, blue for RO–NO_2_ and yellow for RO_2_–NO_2_. To aid interpretation, solid and dashed lines serve as benchmarks: red lines represent NO_3_^−^ clusters, with the solid line for 3-NP/4-NP (plotted together due to very close values) and the dashed line for HNO_3_. Similarly, grey lines represent 
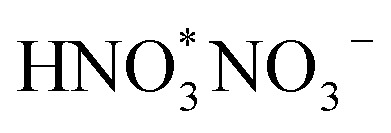
 clusters, with the solid line for 3-NP/4-NP and the dashed line for HNO_3_.

In addition to the RO–NO_2_ clustering results, the binding enthalpies of the corresponding RO_2_–NO species with NO_3_^−^ and 
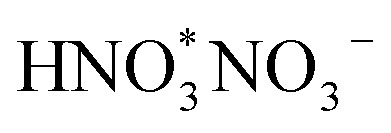
 were also computed and are reported in Section S1, Table S16. The results show that the elusive RO_2_–NO exhibit consistently weaker binding compared to the corresponding RO–NO_2_ species. As the reaction sequence progresses, a gradual increase in binding enthalpy is observed with increasing oxygenation, which results in slower dissociation rates for the more oxidized RO_2_–NO species, allowing time for their rearrangement to RO–NO_2_. However, due to their very short atmospheric lifetimes, direct detection of RO_2_–NO remains unlikely. As a result, the nitrate signals detected in NO_3_^−^-CIMS are predominantly attributed to the more stable RO–NO_2_ products rather than the short-lived RO_2_–NO intermediates. Fig. 5 visualizes the trends in binding enthalpies for RO/RO_2_–NO_2_ species with both NO_3_^−^ and 
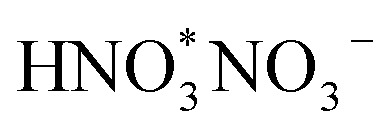
, as a function of oxygen content. For comparison, the binding enthalpies of the benchmark compounds 2-NP, 3-NP, 4-NP, and HNO_3_ are also included.

The plot illustrates that as the oxygen content in RO–NO_2_ and RO_2_–NO_2_ increases their binding affinity with NO_3_^−^ and 
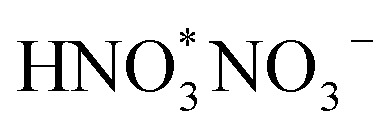
 also strengthens, consistent with the findings of Hyttinen *et al.*^46^ The binding enthalpy of C_6_H_9_O_3_NO_2_ and C_6_H_9_O_4_NO_2,_ with 
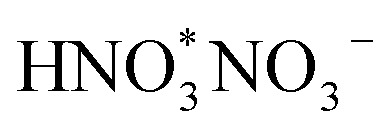
 exceeds HNO_3_ itself, whereas C_6_H_9_O_5_NO_2_, C_6_H_9_O_5_^a^NO_2,_ C_6_H_9_O_6_NO_2_ and C_6_H_9_O_6_^a^NO_2_ exhibit stronger binding surpassing all reference standards (3-nitrophenol, 4-nitrophenol, and HNO_3_). This tendency to make relatively strongly bound clusters with reagent ion dimer indicates potential for misidentification of such species as containing up to two nitrogen atoms, despite having only one. Potentially even higher clusters of the form 
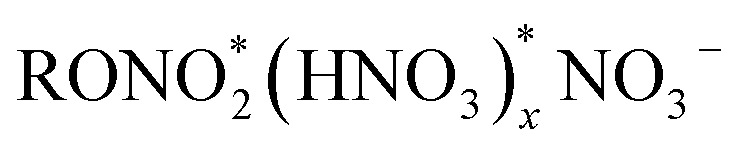
 are thermodynamically stabile and could contribute to the recently described nitrate particle formation in cold environments.^81,82^ Such interferences may arise with other reagent ions as well, highlighting how molecular level insights can be crucial for refining CIMS methodologies, and thereby improve the accuracy of atmospheric chemical measurements, here exemplified for oxygenated organic nitrates. These results underline the importance of integrating molecular-level binding data into atmospheric models and measurement strategies, so that NO_*x*_-driven SOA formation can be more accurately quantified in diverse environments.

## 4. Conclusion

Using computational methods, we have studied RO_2_–NO_*x*_ interactions in early cyclohexene oxidation products. Our results showed that RO_2_ binding with NO and NO_2_ is competitive. Variations in binding strength are observed across different oxidation stages, influenced by the number of oxygen atoms as well as the underlying functional groups and molecular structure. For example, among the secondary peroxy radicals studied, the hydroperoxy-containing C_6_H_9_O_6_^a^–NO and C_6_H_9_O_6_^a^–NO_2_ complexes exhibit relatively higher binding strengths than the corresponding less oxygenated C_6_H_9_O_4_–NO/NO_2_ species. In contrast, the acyl peroxy derived C_6_H_9_O_6_–NO and C_6_H_9_O_6_–NO_2_, despite also containing six oxygen atoms, showed the strongest binding enthalpies among the studied molecules. Notably, C_6_H_11_O_3_–NO/NO_2_ binds more strongly than C_6_H_9_O_4_–NO/NO_2_ despite having one fewer oxygen atom, likely due to the presence of the OH group and associated intramolecular interactions, highlighting the importance of molecular functionality in determining oxidation product stability. These results suggest that the influence of NO_*x*_ on oxidation sequences depends on the specific peroxy radical involved at different oxidation stages.

By analysing the stability and dissociation rate coefficients of RO_2_–NO_*x*_ species, we found that RO_2_–NO intermediates are intrinsically short-lived, dissociating to RO + NO_2_ and promoting radical chain propagation rather than reverting back to RO_2_ + NO. Rearrangement to RO–NO_2_ may compete with dissociation where, its importance is expected to be strongly structure dependent. Accurate treatment of this chemistry would require a more rigorous kinetic and electronic-structure description beyond the scope of the present single-reference DFT approach. For RO_2_–NO_2_ species, two dissociation channels were considered: regeneration of RO_2_ + NO_2_ and fragmentation to RO + NO_3_. The latter pathway is negligible across all non-PAN systems with dissociation to RO_2_ + NO_2_ being the dominant route. For acyl peroxy-NO/NO_2_ systems, NO_2_ or NO_3_ elimination forms acyloxy-type RO radicals that rapidly eliminate CO_2_ to form the C_5_H_9_O_3_ radical. Accurate treatment of this multistep process would require multireference methods to describe the underlying potential energy surface. Thus, while the RO + NO_3_ channel is negligible for non-acyl systems, uncertainties in the dissociation rates of PAN-like species into RO + NO_3_ do not exclude the possibility that such species may contribute to minor NO_3_ formation under certain structural or energetic conditions. However, their quantitative role as NO_3_ sources cannot be established within the present work, motivating further investigation into their potential contribution to atmospheric NO_*x*_ cycling. Within this study, non-PAN RO_2_–NO_2_ species are expected to persist longer than RO_2_–NO, with stability tending to increase with oxidation level and depend sensitively on molecular structure. These species can act as temporary NO_*x*_ reservoirs under high-NO_*x*_ and low-temperature conditions, highlighting the importance of including both NO and NO_2_ chemistry in atmospheric oxidation models.

We also extended our study to evaluate the detection sensitivity of NO_3_^−^-CIMS for the studied molecules by analyzing their binding enthalpies with both NO_3_^−^ and 
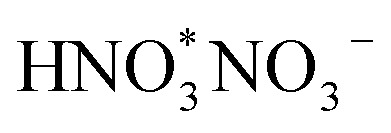
 reagent ions. We used nitrophenol derivatives as reference systems to compare binding enthalpy values, based on prior insights and structural similarity to the studied species. Our calculations showed that 4-nitrophenol, 3-nitrophenol, excluding 2-nitrophenol bind significantly with both NO_3_^−^ and 
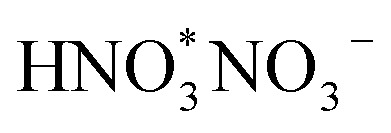
 reagent ions, supporting their suitability comparing with HNO_3_ itself, as reference compounds for evaluating clustering behavior. Less oxidized species such as C_6_H_11_O_2_NO_2_ and C_6_H_11_O_3_NO_2_ exhibited weaker binding with both reagent ions and are thus detected with reduced sensitivity in NO_3_^−^-CIMS experiments. Several of the highest oxygenated species containing C_6_H_9_O_6_NO_2_, C_6_H_9_O_6_^a^NO and C_6_H_9_O_6_^a^NO_2_ demonstrated binding enthalpies very close to those of HNO_3_, 3-nitrophenol and 4-nitrophenol with NO_3_^−^, indicating good experimental detection sensitivity. The C_6_H_9_O_5_NO_2_, C_6_H_9_O_5_^a^NO_2_, C_6_H_9_O_6_NO_2_ and C_6_H_9_O_6_^a^NO_2_ species were found to have greater binding enthalpies than all the reference molecules with both NO_3_^−^ and 
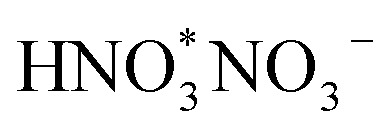
, and thus likely represent species that are detected with maximum sensitivity, often described as “detected at the collision limit”. Almost all RO–NO_2_ species except C_6_H_11_O_2_NO_2_ and C_6_H_11_O_3_NO_2_ were observed to have a stronger binding to 
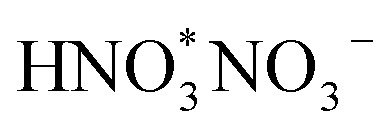
 than HNO_3_
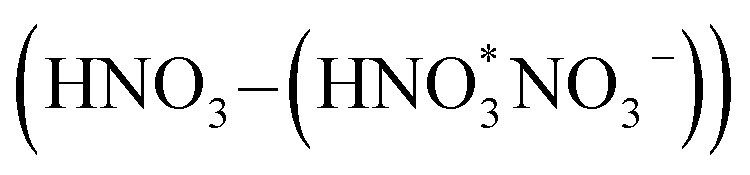
 indicating their strong detection sensitivity. However, species detected as clusters with 
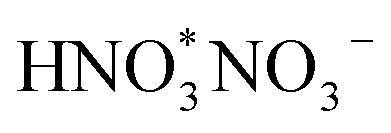
 may easily be misinterpreted in NO_3_^−^-CIMS experiments as having more nitrate groups than they contain. Such misidentifications could bias field observations, leading to skewed estimates of NO_*x*_ involvement and SOA formation potential. This could complicate the interpretation of atmospheric mass spectrometry data especially in high NO_*x*_ environments. Finally, among the most oxygenated nitrates studied, the secondary RO–NO_2_ bind considerably more strongly to 
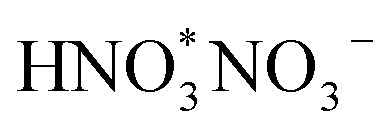
 than the acyl-RONO_2_ analogous, implying their increased capacity to participate in atmospheric particulate formation.

## Author contributions

SJ: conceptualization, formal analysis, investigation, methodology, visualization, writing – original draft; AK: investigation, review & editing; PS: review & editing; SI: conceptualization, formal analysis, review & editing; MR: conceptualization, formal analysis, funding acquisition, methodology, resources, supervision, writing – review & editing.

## Conflicts of interest

There are no conflicts to declare.

## Supplementary Material

EA-006-D5EA00151J-s001

## Data Availability

The data supporting this article have been included in the supplementary information (SI), and the complete set of computational files is available in the Zenodo repository at https://doi.org/10.5281/zenodo.20315985. Supplementary information is available. See DOI: https://doi.org/10.1039/d5ea00151j.
