# Supplementary material for: NO and NO2 reactions with oxygenated peroxy radicals lead to indistinguishable product compositions: computational insights from cyclohexene oxidation in presence of NOx
Source: Environ Sci Atmos. 2026 Jul 14. Online ahead of print. doi: 10.1039/d5ea00151j (PMC13366502; doi:10.1039/d5ea00151j)
Supplement: EA-OLF-D5EA00151J-s001 [file EA-OLF-D5EA00151J-s001.pdf]

This journal is © The Royal Society of Chemistry 2026

Supplementary Information for

NO and NO_2 reactions with oxygenated peroxy radicals lead to indistinguishable product compositions: Computational insights from cyclohexene oxidation in presence of NO_x

Sakshi Jha[1*], Avinash Kumar[1], Prasenjit Seal[1], Siddharth Iyer[1], Matti Rissanen[1,2*]

1. Aerosol Physics Laboratory, Tampere University, 33720 Tampere, Finland

2. Department of Chemistry, University of Helsinki, 00560 Helsinki, Finland

* Corresponding authors

This file includes

Figures S1-S10

Tables S1-S22

Section S1. Computation method analysis, electronic structure details, thermochemical analysis, and Supporting figures

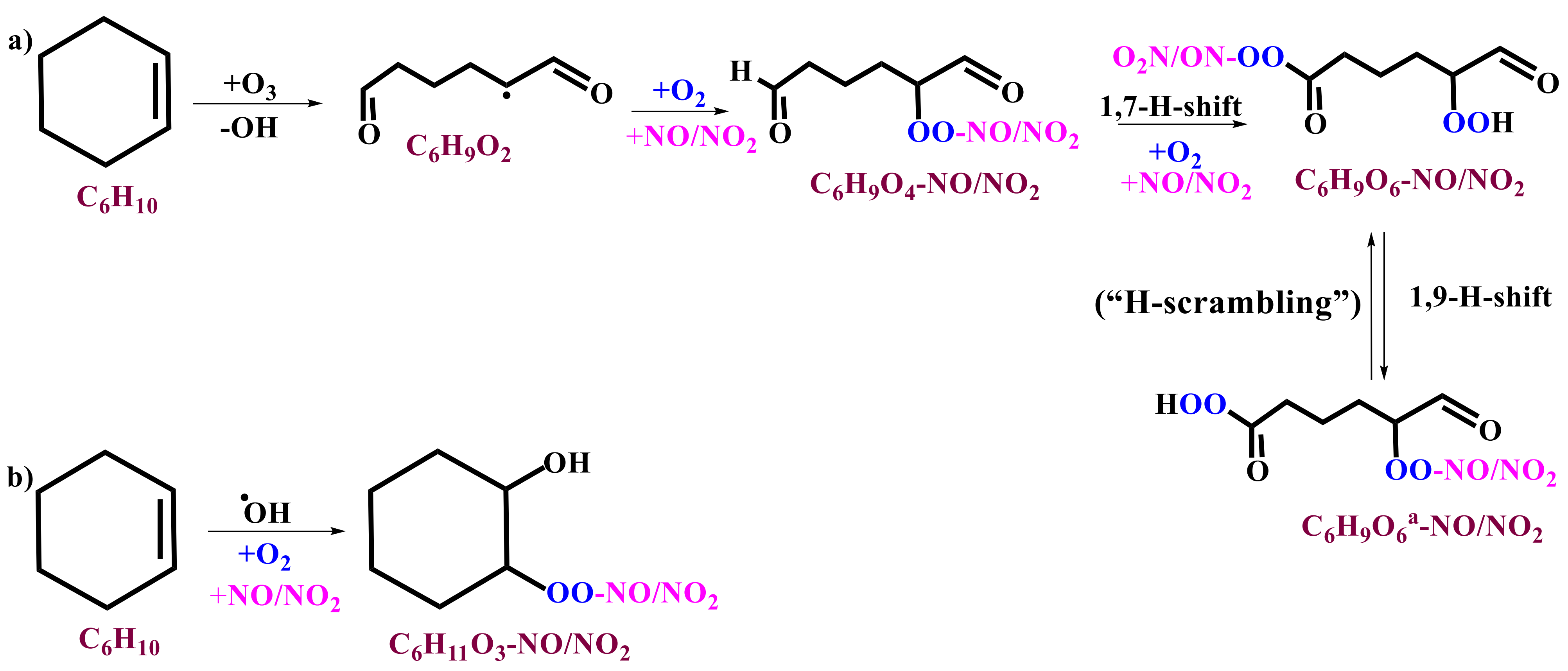

Figure S1. Schematic representation of peroxy radical binding generated from cyclohexene oxidation under different atmospheric conditions: (a) Ozonolysis in the presence of NO and NO_2. (b) Oxidation initiated by OH radical addition in the presence of NO and NO_2.

Table S1. Relative sum of electronic and zero-point energies (Δ (E + ZPE), kcal/mol) of the lowest-energy conformers optimized at the UωB97X-D/aug-cc-pVTZ level of theory, obtained from Spartan MMFF and GOAT (GFN2-xTB) conformer sampling workflows. Spartan energies are taken as the reference (0 kcal/mol).

System	Spartan (kcal/mol)	GOAT (kcal/mol)
$C_6H_{11}O_2$-NO_2	0.00	0.01
$C_6H_{11}O_3$-NO	0.00	0.00
$C_6H_{11}O_3$-NO_2	0.00	0.00

Table S2. Spin expectation values (⟨S^2⟩), coupled-cluster (T_1) diagnostics for the NO, NO_2, NO_3, RO_2, RO_2-NO, RO_2-NO_2, and RO-NO_2 species, and D_1 diagnostics for RO_2, RO, NO, NO_2 and NO_3 species.

SYSTEMS	⟨S^2⟩	T_1 diagnostic	D_1 diagnostic(III)
		**RO_2**	
$C_6H_{11}O_3$	0.750287	0.019184813(II)<br>0.02210884(III)	0.13903804
$C_6H_9O_4$	0.7502794	0.020427424(II)	0.13383729

		0.02231531([III])	
$C_6H_9O_6$	0.7502836	0.018704908([II])<br>0.01967705([III])	0.11585314
$C_6H_9O_6$[a]	0.750265	0.020148899([II])<br>0.02169710([III])	0.13402742
**NO_x**			
NO	0.7502131	0.021948599([II])<br>0.01998831([III])	0.04517856
NO_2	0.7503845	0.023942554([II])<br>0.02370562([III])	0.06003980
NO_3	0.7505814	0.02121737([II])<br>0.03190019([III])	0.11596679
		**RO_2-NO**	
$C_6H_{11}O_3$-NO	0.00([I])	0.015525453	
$C_6H_9O_4$-NO	0.00([I])	0.016877079	
$C_6H_9O_6$-NO	0.00([I])	0.016625781	
$C_6H_9O_6$[a]-NO	0.00([I])	0.017096001	
		**RO**	
$C_6H_{11}O_2$	0.7502116	0.015506914([II])<br>0.01673343([III])	0.08959021
$C_6H_9O_3$	0.7502948	0.01982176([II])<br>0.02101473([III])	0.11478608
$C_6H_9O_5$[a]	0.750255	0.019521982([II])<br>0.02012542([III])	0.11062704
$C_6H_9O_5$	-	-	
		**RO-NO_2**	
$C_6H_{11}O_2$-NO_2	0.00([I])	0.013782998	
$C_6H_9O_3$-NO_2	0.00([I])	0.015618338	
$C_6H_9O_5$-NO_2	0.00([I])	0.015719009	
$C_6H_9O_5$[a]-NO_2	0.00([I])	0.016035233	
		**RO_2-NO_2**	
$C_6H_{11}O_3$-NO_2	0.00([I])	0.014746550	
$C_6H_9O_4$-NO_2	0.00([I])	0.016179872	
$C_6H_9O_6$-NO_2	0.00([I])	0.015951481	
$C_6H_9O_6$[a]-NO_2	0.00([I])	0.016493777	

[I] ⟨S^2⟩ = 0.00 was obtained from unrestricted ωB97X-D/aug-cc-pVTZ calculations using *guess=(mix,always)*, consistent with singlet electronic character

[II] T_1 values calculated are obtained from DLPNO-CCSD(T)/aug-cc-pVTZ.

[III] T_1 and D_1 values calculated from R(O)HF-RCCSD(T)-F12a/VDZ-F12 for open-shell species.

Table S3. T_1 diagnostic values evaluated along the O(peroxy)-N bond dissociation coordinate for $C_6H_{11}O_3$-NO and $C_6H_{11}O_3$-NO_2 systems.

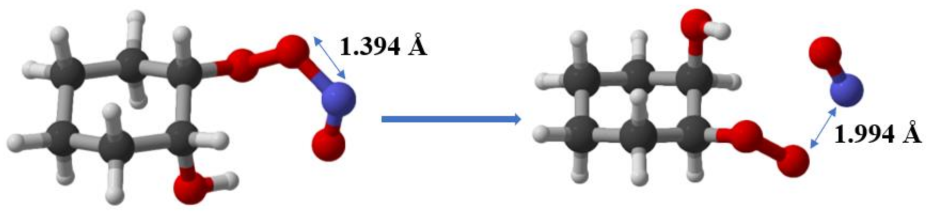

O(peroxy)-N(NO) bond distance (Å)	T_1 diagnostic
1.394	0.015525453
1.544	0.016282653

1.694	0.016643205
1.844	0.017004287
1.994	0.018806446
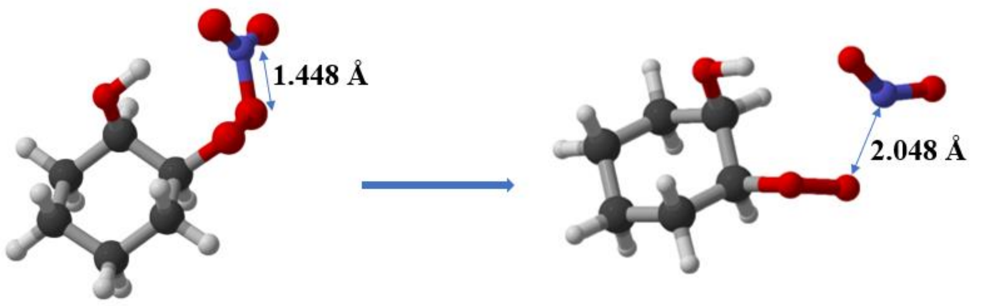	
**O(peroxy)-N(NO) bond distance (Å)**	**T_1 diagnostic**
1.448	0.014746550
1.598	0.015502794
1.748	0.016034562
1.898	0.016149793
2.048	0.016251717

Table S4 Comparison of reaction enthalpies (ΔH, kcal/mol) obtained at the ωB97X-D/aug-cc-pVTZ level and DLPNO-CCSD(T)/aug-cc-pVTZ//ωB97X-D/aug-cc-pVTZ level of theory for the RO_2-NO and RO_2-NO_2 species and RO/RO_2-NO_2 clustered with NO_3^- and HNO_3*NO_3^-.

**RO_2-NO/NO_2**			
**SYSTEMS**	**$\Delta H_{\omega B97X\text{-}D}$ (kcal/mol)**	**$\Delta H_{DLPNO\text{-}CCSD(T)}$ (kcal/mol)**	**$\Delta\Delta H = \Delta H_{DLPNO\text{-}CCSD(T)} - \Delta H_{\omega B97X\text{-}D}$ (kcal/mol)**
$C_6H_{11}O_3NO$	22.18495319	25.68681769	3.501864507
$C_6H_9O_4NO$	21.23427705	24.84812697	3.613849917
$C_6H_9O_6NO$	29.64478018	33.340498	3.695717818
$C_6H_9O_6{}^aNO$	21.86304107	26.07656678	4.213525716
$C_6H_{11}O_3NO_2$	19.96357133	24.70982196	4.746250637
$C_6H_9O_4NO_2$	19.39504817	24.10827193	4.713223755
$C_6H_9O_6NO_2$	26.08303909	30.85852435	4.775485252
$C_6H_9O_6{}^aNO_2$	19.77469112	25.06051641	5.285825298
**(RO/RO_2-NO_2)-NO_3^-**			
$C_6H_{11}O_3NO_2$-NO_3^-	19.62471647	21.11516781	1.490451342
$C_6H_{11}O_2NO_2$-NO_3^-	21.22423691	22.32313902	1.098902112
$C_6H_9O_4NO_2$-NO_3^-	25.02505892	26.58147336	1.556414442
$C_6H_9O_3NO_2$-NO_3^-	25.58291442	26.49157543	0.908661006
$C_6H_9O_6NO_2$-NO_3^-	28.73865718	30.13825371	1.399596526
$C_6H_9O_5NO_2$-NO_3^-	28.59433011	30.08537339	1.491043281
$C_6H_9O_6{}^aNO_2$-NO_3^-	30.42100881	32.50006345	2.079054636
$C_6H_9O_5{}^aNO_2$-NO_3^-	32.04437459	34.27430233	2.22992774
**(RO/RO_2-NO_2) - (HNO_3*NO_3^-)**			
$C_6H_{11}O_3NO_2$-HNO_3*NO_3^-	12.19751994	13.9242319	1.726711963
$C_6H_{11}O_2NO_2$-HNO_3*NO_3^-	14.02670868	15.53649371	1.509785029
$C_6H_9O_4NO_2$-HNO_3*NO_3^-	19.26703634	21.57773617	2.310699839
$C_6H_9O_3NO_2$-HNO_3*NO_3^-	19.79728144	21.41003385	1.612752414
$C_6H_9O_6NO_2$-HNO_3*NO_3^-	20.68646169	22.84988219	2.163420493
$C_6H_9O_5NO_2$-HNO_3*NO_3^-	20.80317837	23.07636105	2.273182685
$C_6H_9O_6{}^aNO_2$-HNO_3*NO_3^-	20.59359036	24.38622575	3.792635387
$C_6H_9O_5{}^aNO_2$-HNO_3*NO_3^-	22.57902884	25.67300648	3.093977639

Table S5. Binding energies (ΔE, kcal/mol) for the $C_2H_5O_2NO$ and $C_2H_5O_2NO_2$ species, calculated relative to the separated fragments ($C_2H_5O_2$ + NO/NO_2) using various levels of theory.

Methods	$C_2H_5O_2NO$ ΔE (kcal/mol)	$C_2H_5O_2NO_2$ ΔE (kcal/mol)
DLPNO-CCSD(T)/aug-cc-pVTZ//ωB97X-D/aug-cc-pVTZ	24.97	25.40
R(O)HF–RCCSD(T)-F12a/VDZ-F12//ωB97X-D/aug-cc-pVTZ	27.03	26.91
canonical CCSD(T)//ωB97X-D/aug-cc-pVTZ	25.69	25.80
DLPNO-CCSD(T))/aug-cc-pVTZ//M06-2X/aug-cc-pVTZ	24.99	25.45

Table S6. Binding enthalpies (ΔH, kcal/mol) for the $C_2H_5O_2NO$ and $C_2H_5O_2NO_2$ species, calculated relative to the separated fragments ($C_2H_5O_2$ + NO/NO_2) using various levels of theory at 298.15K.

Methods	$C_2H_5O_2NO$ ΔH (kcal/mol)	$C_2H_5O_2NO_2$ ΔH (kcal/mol)
DLPNO-CCSD(T)// ωB97X-D/aug-cc-pVTZ	23.33	22.82
R(O)HF–RCCSD(T)-F12a/VDZ-F12) //ωB97X-D/aug-cc-pVTZ	25.39	24.33
canonical CCSD(T)//ωB97X-D/aug-cc-pVTZ	24.05	23.22
DLPNO-CCSD(T)//M06-2X/aug-cc-pVTZ	23.32	22.81

Table S7. Reaction Gibbs free energies (ΔG, kcal/mol) for $C_2H_5O_2NO$ and $C_2H_5O_2NO_2$ species into their respective product channels, calculated using various levels of theory at 298.15K.

Methods	$C_2H_5O_2NO$ ΔG (kcal/mol)		$C_2H_5O_2NO_2$ ΔG (kcal/mol)	
	$C_2H_5O_2NO \rightarrow C_2H_5O_2$ + NO	$C_2H_5O_2NO \rightarrow C_2H_5O$ + NO_2	$C_2H_5O_2NO_2 \rightarrow C_2H_5O_2 + NO_2$	$C_2H_5O_2NO_2 \rightarrow C_2H_5O$ + NO3
DLPNO-CCSD(T)//ωB97X-D/aug-cc-pVTZ	11.82	2.20	9.57	27.43
R(O)HF–RCCSD(T)-F12a/VDZ-F12 //ωB97X-D/aug-cc-pVTZ	13.88	6.05	11.08	28.93
canonical CCSD(T)//ωB97X-D/aug-cc-pVTZ	12.54	2.38	9.97	25.81
DLPNO-CCSD(T)//M06-2X/aug-cc-pVTZ	11.75	-*	9.97	-*

*For the M06-2X/aug-cc-pVTZ calculations, the C_2H_5O (alkoxy) radical could not be optimized to a true minimum and showed a significant imaginary frequency, in line with previous studies.[1]

Table S8. Binding enthalpies (ΔH) and Gibbs free energies (ΔG) (kcal/mol) for $C_6H_{11}O_3$-NO/NO_2 and $C_6H_9O_4$-NO/NO_2 species calculated using ωB97X-D/aug-cc-pVTZ and M06-2X/aug-cc-pVTZ levels of theory at 298.15K.

SYSTEMS	RO_2-NO				RO_2-NO_2			
	ωB97X-D/aug-cc-pVTZ		M06-2X/aug-cc-pVTZ		ωB97X-D/aug-cc-pVTZ		M06-2X/aug-cc-pVTZ	
	ΔH	ΔG	ΔH	ΔG	ΔH	ΔG	ΔH	ΔG
$C_6H_{11}O_3$	22.18	-10.34	23.72	-11.29	19.96	-6.33	24.55	-10.83
$C_6H_9O_4$	21.23	-9.50	22.36	-10.75	19.40	-6.06	22.72	-10.10

Table S9 Binding enthalpies (ΔH) and Gibbs free energies (ΔG) (kcal/mol) for $C_6H_{11}O_3$-NO/NO_2 and $C_6H_9O_4$-NO/NO_2 species calculated using DLPNO-CCSD(T)/ aug-cc-pVTZ //ωB97X-D/aug-cc-pVTZ and DLPNO-CCSD(T)/ aug-cc-pVTZ//M06-2X/aug-cc-pVTZ levels of theory at 298.15K.

SYSTEMS	RO_2-NO	RO_2-NO_2

	DLPNO-CCSD(T)/ aug-cc-pVTZ //ωB97X-D/aug-cc-pVTZ		DLPNO-CCSD(T)/ aug-cc-pVTZ//M06-2X/aug-cc-pVTZ		DLPNO-CCSD(T)/ aug-cc-pVTZ //ωB97X-D/aug-cc-pVTZ		DLPNO-CCSD(T)/ aug-cc-pVTZ//M06-2X/aug-cc-pVTZ	
	ΔH	ΔG	ΔH	ΔG	ΔH	ΔG	ΔH	ΔG
$C_6H_{11}O_3$	25.69	-13.84	25.18	-12.76	24.71	-11.07	24.23	-10.51
$C_6H_9O_4$	24.85	-13.11	24.56	-12.95	24.11	-10.78	23.44	-10.83

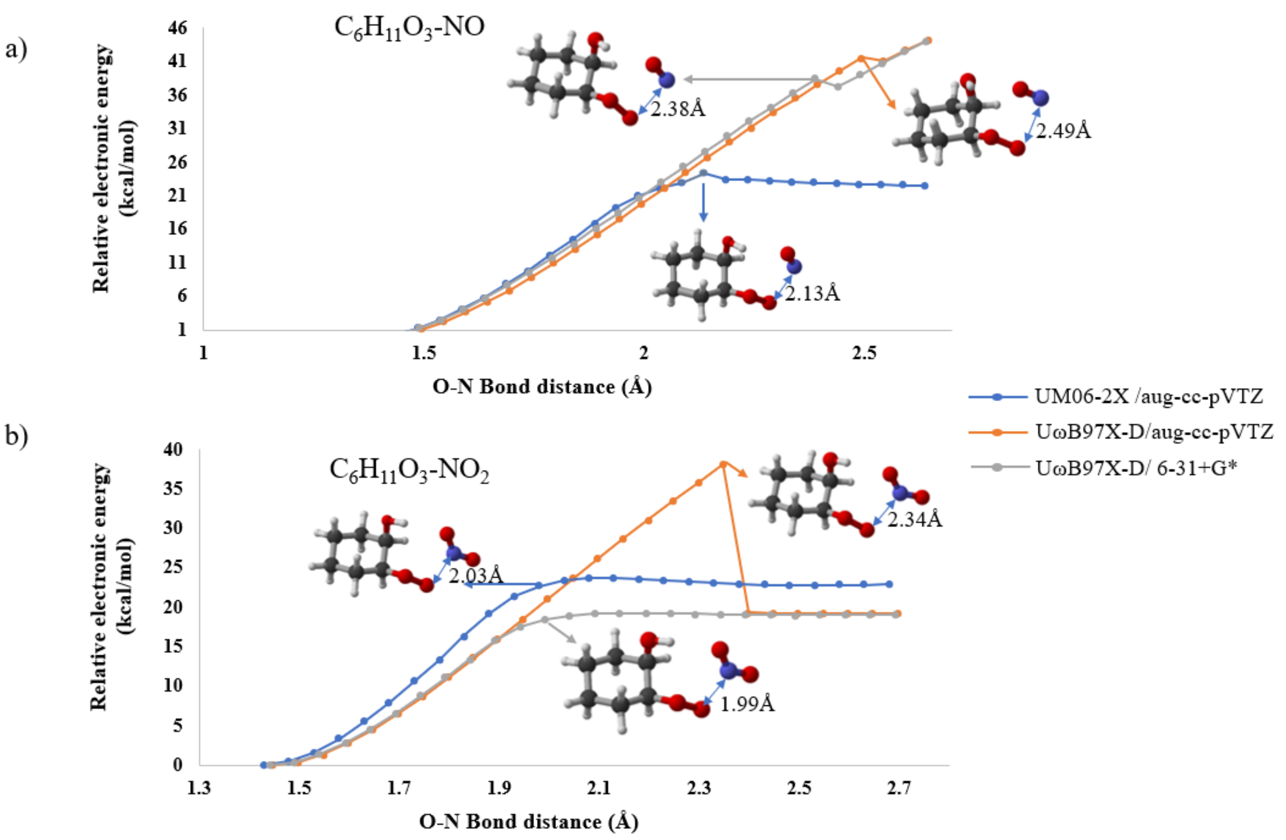

Figure S2. Relaxed scan profiles of the $C_6H_{11}O_3NO$ and $C_6H_{11}O_3NO_2$ bond dissociation are shown as relative electronic energies (kcal/mol) computed at the UωB97X-D/aug-cc-pVTZ , UM06-2X/aug-cc-pVTZ and UωB97X-D/6-31+G* level of theory, plotted as a function of the O(peroxy)-N(NO_2) bond distance. The orange curves correspond to UωB97X-D/aug-cc-pVTZ, the blue curves to UM06-2X/aug-cc-pVTZ, and the grey curves to UωB97X-D/6-31+G* method. a) For $C_6H_{11}O_3NO$, the relaxed scan exhibits a monotonic increase in energy upon elongation of the O-N distance, with no indication of a stationary minimum or maximum along the dissociation coordinate. This behaviour was observed over O-N distances of 1.39 to 2.49 Å, 1.38 to 2.13 Å and 1.38 to 2.38 Å using the UωB97X-D/aug-cc-pVTZ, UM06-2X/aug-cc-pVTZ, and UωB97X-D/6-31+G* methods respectively. b) For $C_6H_{11}O_3NO_2$, a similar monotonic increase in energy was observed along the dissociation coordinate over O-N distances of 1.44 to 2.34 Å, 1.43 to 2.03 Å and 1.44 to 1.99 Å using the UωB97X-D/aug-cc-pVTZ, UM06-2X/aug-cc-pVTZ, and UωB97X-D/6-31+G* methods respectively.

Note: (1) For $C_6H_{11}O_3$-NO_2 at the UωB97X-D/aug-cc-pVTZ level of theory, a rapid decrease in energy is observed after O-N separations (1.44 to 2.34 Å) where the bond is effectively dissociated. The corresponding geometries suggest that this behaviour arises from the interactions between NO_2 and the OH group of the RO_2 fragment, rather than passage over a transition state, as attempts to locate a corresponding transition structure were unsuccessful.

(2) Both UωB97X-D/aug-cc-pVTZ and UM06-2X/aug-cc-pVTZ scans exhibited method-dependent differences with increasing O-N separations. Depending on the system and computational method, spin contamination began to increase at extended O-N separations. For the UM06-2X/aug-cc-pVTZ scans, $\langle S^2 \rangle$ values increased from ~0.0007 to 0.726 for $C_6H_{11}O_3NO$ beyond an O-N distance of ~1.88 Å and from ~0.0005 to 0.6129 for $C_6H_{11}O_3NO_2$ beyond ~1.98 Å. The corresponding UωB97X-D/aug-cc-pVTZ and UωB97X-D/6-31+G* scans showed substantially smaller post-annihilation $\langle S^2 \rangle$ deviations for $C_6H_{11}O_3NO_2$ (~0.0456 - 0.0636 beyond ~1.79 Å and ~0.002 - 0.0599 beyond ~1.89 Å, respectively), while the $C_6H_{11}O_3NO$ scans showed no spin contamination. These observations suggest that the long-range dissociation region becomes electronically sensitive to the chosen method and highlight the limitations of constrained single-reference DFT scans for rigorously determining minima or barriers on the dissociation potential energy surface.

Table S10. Reaction Gibbs free energy (ΔG, kcal/mol) for RO_2-NO.

**RO2NO**	RO_2-NO → RO_2 + NO		RO_2-NO → RO + NO_2		RO_2-NO → RO-NO_2	
	**†ΔG (kcal/mol) without Hinder Rotor**	**†ΔG(kcal/mol) with Hinder Rotor**	**†ΔG (kcal/mol) without Hinder Rotor**	**†ΔG(kcal/mol) with Hinder Rotor**	**†ΔG (kcal/mol) without Hinder Rotor**	**†ΔG(kcal/mol) with Hinder Rotor**
$C_6H_{11}O_3NO$	-13.84070279	-13.84489918	-2.090486415	-2.094682801	-25.95414675	-25.94662818
$C_6H_9O_4NO$	-13.11308116	-13.11310371	-1.713174104	-1.713196656	-25.96948533	-25.97840137
$C_6H_9O_6NO$	-21.8225703	-21.8223964	-	-	-20.6104545	-20.61094135
$C_6H_9O_6{}^aNO$	-14.49275064	-14.49279167	-2.670430523	-2.670471546	-24.29468728	-24.29403609

† All values were obtained at the DLPNO-CCSD(T)/aug-cc-pVTZ//ωB97X-D/aug-cc-pVTZ level of theory at 298.15 K along the principal dissociation and isomerization pathways. ΔG values are reported both with and without one-dimensional hindered-rotor corrections for low-frequency torsional modes.

Table S11. Reaction Gibbs free energy (ΔG, kcal/mol) for RO_2-NO_2.

**RO_2NO_2**	RO_2-NO_2 → RO_2 + NO_2		RO_2-NO_2 → RO + NO_3	
	**†ΔG (kcal/mol) without Hinder Rotor**	**†ΔG(kcal/mol) with Hinder Rotor**	**†ΔG (kcal/mol) without Hinder Rotor**	**†ΔG (kcal/mol) with Hinder Rotor**
$C_6H_{11}O_3NO_2$	-11.07405139	-11.07624962	-22.52028704	-26.817556
$C_6H_9O_4NO_2$	-10.7755882	-10.79096228	-22.57213317	-26.88257799
$C_6H_9O_6NO_2$	-17.5452934	-17.53034694	-	-
$C_6H_9O_6{}^aNO_2$	-11.60421342	-11.59804574	-22.97834532	-27.26724838

† All values were obtained at the DLPNO-CCSD(T)/aug-cc-pVTZ//ωB97X-D/aug-cc-pVTZ level of theory at 298.15 K along the dissociation and isomerization pathways. ΔG values are reported both with and without one-dimensional hindered-rotor corrections for low-frequency torsional modes.

Table S12. Reaction Gibbs free energy (ΔG kcal/mol) for the reversion, dissociation, and isomerization pathways of RO_2-NO and RO_2-NO_2 obtained at the ωB97X-D/aug-cc-pVTZ level of theory at 298.15 K.

**RO_2**	**RO_2-NO**			**RO_2-NO_2**	
	**ΔG for RO_2 + NO (kcal/mol)**	**ΔG for RO + NO_2 kcal/mol**	**ΔG for RO-NO_2 kcal/mol**	**ΔG for RO_2 + NO_2 (kcal/mol)**	**ΔG for RO + NO_3 (kcal/mol)**
$C_6H_{11}O_3$	10.38	-6.03	-30.26	6.32	16.96
$C_6H_9O_4$	9.49	-7.64	-29.85	6.06	15.92
$C_6H_9O_6$	18.12	-6.51[I]<br>-31.43[II]	-24.40	12.76	15.13[I]<br>-9.78[II]
$C_6H_9O_6{}^a$	10.27	-7.39	-28.68	6.31	15.64

[I]ΔG values correspond to constrained optimizations performed at the ωB97X-D/aug-cc-pVTZ level, where a C-C bond is constrained that prevents the direct elimination of CO_2 during RO optimization, thereby leading to RO + NO_2 and RO + NO_3, respectively.

[II]ΔG values correspond to the unconstrained case, allowing spontaneous CO_2 loss, computed at the ωB97X-D/aug-cc-pVTZ level. In this pathway, RO_2-NO dissociates R + CO_2 + NO_2, while RO_2NO_2 dissociates to R + CO_2 + NO_3.

Table S13. Sensitivity of calculated dissociation rate constants (k_{diss}) for RO_2-NO intermediates to the assumed collision-limit rate coefficient k_{coll} (2 x 10^{-11} - 2 x 10^{-12} cm^3 $molecule^{-1}$ s^{-1}) and the pre-exponential factor (A) used in MESMER.

**k_{coll} = 2 x 10^{-11} cm^3 $molecule^{-1}$ s^{-1}; A = 2 x 10^{-11} cm^3 $molecule^{-1}$ s^{-1}**						
RO_2-NO	RO_2-NO → RO_2 +NO			RO_2-NO → RO +NO_2		
	${}^{b}k_{diss}^{Detailed\ Balence}$	${}^{c}k_{diss}^{MESMER-HO}$	${}^{d}k_{diss}^{MESMER-HR}$	${}^{b}k_{diss}^{Detailed\ Balence}$	${}^{c}k_{diss}^{MESMER-HO}$	${}^{d}k_{diss}^{MESMER-HR}$
$C_6H_{11}O_3NO$	3.51 x 10^{-02}	2.04 x 10^{-02}	3.56 x 10^{-03}	1.44 x 10^{+07}	6.89 x 10^{+05}	4.04 x 10^{+05}
$C_6H_9O_4NO$	1.20 x 10^{-01}	3.66 x 10^{-02}	1.42 x 10^{-03}	2.72 x 10^{+07}	1.77 x 10^{+06}	3.18 x 10^{+05}
$C_6H_9O_6NO$	4.94 x 10^{-08}	1.11 x 10^{-08}	-	-	-	-
$C_6H_9O_6{}^aNO$	1.17 x 10^{-02}	1.95 x 10^{-03}	-	5.40 x 10^{+06}	6.70 x 10^{+05}	-
**k_{coll} = 2 x 10^{-12} cm^3 $molecule^{-1}$ s^{-1}; A = 2 x 10^{-12} cm^3 $molecule^{-1}$ s^{-1}**						

$C_6H_{11}O_3NO$	3.51 x 10^{-03}	2.04 x 10^{-03}	3.56 x 10^{-04}	1.44 x 10^{+06}	2.74 x 10^{+05}	1.07 x 10^{+05}
$C_6H_9O_4NO$	1.20 x 10^{-02}	3.67 x 10^{-03}	1.42 x 10^{-04}	2.72 x 10^{+06}	5.77 x 10^{+05}	3.59 x 10^{+04}
$C_6H_9O_6NO$	4.94 x 10^{-09}	1.11 x 10^{-09}		-		
$C_6H_9O_6{}^aNO$	1.17 x 10^{-03}	1.95 x 10^{-04}		5.40 x 10^{+05}	1.01 x 10^{+05}	

Table S14. Sensitivity of calculated dissociation rate constants (k_{diss}) for RO_2-NO_2 intermediates to the assumed collision-limit rate coefficient k_{coll} (2 x 10^{-11} - 2 x 10^{-12} cm^3 molecule^{-1} s^{-1}) and the pre-exponential factor (A) used in MESMER.

**k_{coll} = 2 x 10^{-11} cm^3 molecule^{-1} s^{-1}; A = 2 x 10^{-11} cm^3 molecule^{-1} s^{-1}**						
RO_2-NO_2	RO_2-NO_2 → RO_2 + NO_2			RO_2-NO_2 → RO + NO_3		
	${}^bk_{diss}^{Detailed\ Balence}$	${}^ck_{diss}^{MESMER-HO}$	${}^dk_{diss}^{MESMER-HR}$	${}^bk_{diss}^{Detailed\ Balence}$	${}^ck_{diss}^{MESMER-HO}$	${}^dk_{diss}^{MESMER-HR}$
$C_6H_{11}O_3NO_2$	3.74 x 10^{+00}	1.93 x 10^{+00}	1.12 x 10^{+00}	1.08 x 10^{-11}	6.09 x 10^{-12}	3.51 x 10^{-12}
$C_6H_9O_4$ NO_2	6.19 x 10^{+00}	1.62 x 10^{+00}	3.31 x 10^{-02}	9.92 x 10^{-12}	2.93 x 10^{-12}	5.98 x 10^{-14}
$C_6H_9O_6$ NO_2	6.75 x 10^{-05}	1.21 x 10^{-05}	-	-	-	-
$C_6H_9O_6{}^a$ NO_2	1.53 x 10^{+00}	2.16 x 10^{-01}	3.49 x 10^{-03}	5.00 x 10^{-12}	8.33 x 10^{-13}	1.35 x 10^{-14}
**k_{coll} = 2 x 10^{-12} cm^3 molecule^{-1} s^{-1}; A = 2 x 10^{-12} cm^3 molecule^{-1} s^{-1}**						
$C_6H_{11}O_3NO_2$	3.74 x 10^{-01}	1.96 x 10^{-01}	1.13 x 10^{-01}	1.08 x 10^{-12}	6.09 x 10^{-13}	3.51 x 10^{-13}
$C_6H_9O_4$ NO_2	6.19 x 10^{-01}	1.63 x 10^{-01}	3.31 x 10^{-03}	9.91 x 10^{-13}	2.93 x 10^{-13}	5.98 x 10^{-15}
$C_6H_9O_6$ NO_2	6.75 x 10^{-06}	1.21 x 10^{-06}	-	-	-	-
$C_6H_9O_6{}^a$ NO_2	1.53 x 10^{-01}	2.16 x 10^{-02}	3.49 x 10^{-04}	4.99 x 10^{-13}	8.33 x 10^{-14}	1.35 x 10^{-15}

Table S15. Dissociation rates ($k_{diss}^{Detailed\ Balence}$) of RO_2-NO and RO_2-NO_2 in s^{-1}.

**RO_2-NO**	**RO_2-NO → RO +NO_2**		**RO_2-NO_2 → RO +NO_3**	
	**ΔG kcal/mol**	**††$k_{diss}^{Detailed\ Balence}$**	**ΔG kcal/mol**	**††$k_{diss}^{Detailed\ Balence}$**
$C_6H_{11}O_3NO$	6.029733981	1.28 x 10^{14}	-16.96219578	1.81 x 10^{-3}
$C_6H_9O_4NO$	7.642432111	1.95 x 10^{15}	-15.92366838	1.04 x 10^{-2}
$C_6H_9O_6NO$	6.51354342	2.91 x 10^{14}	-15.13363455	3.95 x 10^{-2}
$C_6H_9O_6{}^aNO$	7.397703601	1.29 x 10^{15}	-15.64442688	1.67 x 10^{-2}

††The reported values were derived via detailed balance from the Gibbs free energy changes (ΔG) obtained at the ωB97X-D/aug-cc-pVTZ level of theory at 298.15 K, using a representative collision rate of 2 × 10^{-10} cm^3 molecule^{-1} s^{-1}.

Table S16 Adduct binding enthalpy (ΔH) of RO_2-NO molecules HNO_3 with NO_3^- and HNO_3*NO_3^- in kcal/mol. calculated at the DLPNO-CCSD(T)/ aug-cc-pVTZ //ωB97xD/aug-cc-pVTZ level of theory at 298.15 K.

**SYSTEMS**	**ΔH (kcal/mol) System-NO_3^-**	**ΔH (kcal/mol) System-(HNO3*NO_3^-)**
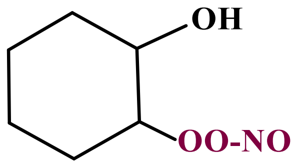<br>**$C_6H_{11}O_3NO$**	18.53	13.16

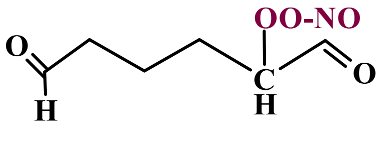 $C_6H_9O_4NO$	24.75	19.98
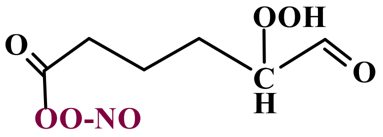 $C_6H_9O_6NO$	28.24	20.49
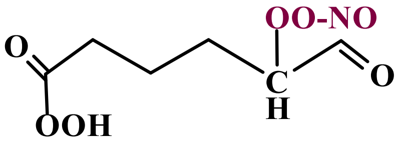 $C_6H_9O_6{}^aNO$	30.40	20.43

Table S17. Zero-point energy (ZPE), Enthalpy (H), Gibbs free energy (G), Electronic energy (E), and single-point electronic energy (E(CCSD(T))) in Hartree computed at DLPNO-CCSD(T)/aug-cc-pVTZ//ωB97X-D/aug-cc-pVTZ level of theory for peroxy radicals, alkoxy radical, NO_3, NO, NO_2, and their corresponding products.

Systems	ZPE	H (Hartree)	G (Hartree)	E (Hartree)	E(CCSD(T)) (Hartree)	Corrected H[#] (Hartree)	Corrected G[##] (Hartree)	Corrected ZPE[###] (Hartree)
NO	-129.892504	-129.889199	-129.91248	-129.8971159	-129.724856052	-129.716939179	-129.740220179	-129.720244179
NO_2	-205.079437	-205.075568	-205.103418	-205.0884872	-204.8137294	-204.800810201	-204.8286602	-204.8046792
NO_3	-280.22501	-280.22046	-280.251324	-280.2368114	-279.858777	-279.842425661	-279.8732897	-279.8469757
CO_2	-188.586595	-188.583046	-188.607275	-188.5984729	-188.3384225	-188.322995611	-188.3472246	-188.3265446
$C_5H_9O_3$	-421.337758	-421.327321	-421.372799	-421.4741469	-420.8256105	-420.678784613	-420.7242626	-420.6892216
$C_6H_{11}O_2$	-385.506438	-385.498051	-385.538363	-385.6738214	-385.0442625	-384.868492080	-384.9088041	-384.8768791
$C_6H_{11}O_3$	-460.669721	-460.660373	-460.703216	-460.8424535	-460.1177565	-459.935675927	-459.9785189	-459.9450239
$C_6H_9O_4$	-534.676824	-534.664812	-534.716169	-534.8250508	-534.0095545	-533.849315701	-533.9006727	-533.8613277
$C_6H_9O_3$	-459.515341	-459.504412	-459.552548	-459.6582028	-458.9360544	-458.782263600	-458.8303996	-458.7931926
$C_6H_9O_6$	-685.051868	-685.038581	-685.092035	-685.2105375	-684.2068879	-684.034931451	-684.0883855	-684.0482185
$C_6H_9O_5$_CONSTRAINED	-609.901192	-609.888641	-609.940362	-610.0544525	-	-	-	-
$C_6H_9O_6{}^a$	-685.064799	-685.050923	-685.107942	-685.2229321	-684.2177783	-684.045769145	-684.1027881	-684.0596451
$C_6H_9O_5{}^a$	-609.903689	-609.890712	-609.945174	-610.0558074	-609.1438216	-608.978726203	-609.0331882	-608.9917032
$C_6H_{11}O_3NO$	-590.596257	-590.584926	-590.632172	-590.7774535	-589.8860772	-589.693549687	-589.7407957	-589.7048807
$C_6H_{11}O_2NO_2$	-590.644546	-590.633551	-590.680408	-590.8282334	-589.9299817	-589.735299283	-589.7821563	-589.7462943
$C_6H_9O_4NO$	-664.601795	-664.58785	-664.643787	-664.7586421	-663.776645	-663.605852921	-663.6617899	-663.6197979
$C_6H_9O_3NO_2$	-664.651688	-664.638525	-664.691346	-664.8115354	-663.8233644	-663.650353961	-663.703175	-663.663517
$C_6H_9O_6NO$	-814.99033	-814.975022	-815.033402	-815.1576205	-813.9876007	-813.805002135	-813.8633821	-813.8203101
$C_6H_9O_5NO_2$	-815.029191	-815.014201	-815.072293	-815.1986552	-814.0225892	-813.838135010	-813.896227	-813.853125
$C_6H_9O_6{}^aNO$	-814.990771	-814.974963	-815.036803	-815.1575379	-813.9868389	-813.804264010	-813.866104	-813.820072
$C_6H_9O_5{}^aNO_2$	-815.038971	-815.024002	-815.082519	-815.2091435	-814.0314446	-813.846303088	-813.9048201	-813.8612721
$C_6H_{11}O_3NO_2$	-665.779658	-665.767755	-665.816718	-665.9668976	-664.9750064	-664.775863766	-664.8248268	-664.7877668
$C_6H_9O_4NO_2$	-739.785884	-739.771288	-739.829248	-739.9485259	-738.8657828	-738.688544908	-738.7465049	-738.7031409
$C_6H_9O_6NO_2$	-890.171557	-890.155715	-890.215803	-890.3449775	-889.0741804	-888.884917878	-888.9450059	-888.9007599
$C_6H_9O_6{}^aNO_2$	-890.174363	-890.158004	-890.221429	-890.3471766	-889.0756885	-888.886515852	-888.9499409	-888.9028749

[#]Corrected H = (Sum of electronic and thermal enthalpies(H)) $_{DFT}$ - (Electronic Energy $_{DFT}$(E) - Single-point Electronic Energy $_{DLPNO\text{-}CCSD(T)}$(E(CCSD(T)))
[##]Corrected G = (Sum of electronic and thermal Free Energies(G)) $_{DFT}$ - (Electronic Energy $_{DFT}$(E) - Single-point Electronic Energy $_{DLPNO\text{-}CCSD(T)}$(E(CCSD(T)))
[###] Corrected ZPE = (Sum of electronic and zero-point energies (ZPE)) $_{DFT}$ - (Electronic Energy $_{DFT}$(E) - Single-point Electronic Energy $_{DLPNO\text{-}CCSD(T)}$(E(CCSD(T)))

Table S18. Zero-point energy (ZPE), Enthalpy (H), Gibbs free energy (G), Electronic energy (E), and single-point electronic energy (E(CCSD(T))) in Hartree computed at DLPNO-CCSD(T)/aug-cc-pVTZ//ωB97X-D/aug-cc-pVTZ level of theory including hindered-rotor (HR) corrections for RO_2-NO, RO_2-NO_2 and $RONO_2$.

**Systems**	**ZPE**	**H (Hartree)**	**G (Hartree)**	**E (Hartree)**	**E(CCSD(T)) (Hartree)**	**Corrected H[#] (Hartree)**	**Corrected G[##] (Hartree)**	**Corrected ZPE[###] (Hartree)**
$C_6H_{11}O_3NO$	-590.59626	-590.58493	-590.632177	-590.7774536	-589.8860789	-589.6935534	-589.7408024	-589.7048854
$C_6H_{11}O_2NO_2$	-590.644542	-590.63355	-590.680409	-590.8282335	-589.9299755	-589.735287	-589.782151	-589.746284
$C_6H_9O_4NO$	-664.601795	-664.58785	-664.643787	-664.7586421	-663.776645	-663.605853	-663.66179	-663.619798
$C_6H_9O_3NO_2$	-664.651694	-664.63853	-664.69137	-664.8115354	-663.8233546	-663.6503452	-663.7031892	-663.6635132
$C_6H_9O_6NO$	-814.99033	-814.97502	-815.033402	-815.1576205	-813.9876004	-813.8050019	-813.8633819	-813.8203099
$C_6H_9O_5NO_2$	-815.029191	-815.0142	-815.072293	-815.1986552	-814.0225897	-813.8381355	-813.8962275	-813.8531255
$C_6H_9O_6^aNO$	-814.990771	-814.97496	-815.036803	-815.1575379	-813.986839	-813.8042641	-813.8661041	-813.8200721
$C_6H_9O_5^aNO_2$	-815.038971	-815.024	-815.082518	-815.2091435	-814.0314446	-813.8463031	-813.9048191	-813.8612721
$C_6H_{11}O_3NO_2$	-665.77966	-665.76776	-665.816714	-665.9668977	-664.975014	-664.7758733	-664.8248303	-664.7877763
$C_6H_9O_4NO_2$	-739.785888	-739.77129	-739.829278	-739.9485259	-738.8657773	-738.6885404	-738.7465294	-738.7031394
$C_6H_9O_6NO_2$	-890.171549	-890.15571	-890.215773	-890.3449777	-889.0741867	-888.8849211	-888.9449821	-888.9007581
$C_6H_9O_6^aNO_2$	-890.174363	-890.15801	-890.221413	-890.3471766	-889.0756947	-888.886523	-888.949931	-888.902881

[#]Corrected H = (Sum of electronic and thermal enthalpies(H)) $_{DFT}$ - (Electronic Energy $_{DFT}$(E) - Single-point Electronic Energy $_{DLPNO\text{-}CCSD(T)}$(E(CCSD(T)))
[##]Corrected G = (Sum of electronic and thermal Free Energies(G)) $_{DFT}$ - (Electronic Energy $_{DFT}$(E) - Single-point Electronic Energy $_{DLPNO\text{-}CCSD(T)}$(E(CCSD(T)))
[###] Corrected ZPE = (Sum of electronic and zero-point energies (ZPE)) $_{DFT}$ - (Electronic Energy $_{DFT}$(E) - Single-point Electronic Energy $_{DLPNO\text{-}CCSD(T)}$(E(CCSD(T)))

Table S19. Enthalpy (H), Gibbs free energy (G), electronic energy (E), and single-point electronic energy (E(CCSD(T))) in Hartree computed at the DLPNO-CCSD(T)/aug-cc-pVTZ//ωB97X-D/aug-cc-pVTZ level of theory for clusters formed with nitrate ion, and nitric acid - nitrate reagent dimers ion in Hartree at 298.15 K.

**Systems**	**H (Hartree)**	**G (Hartree)**	**E (Hartree)**	**E(CCSD(T)) (Hartree)**	**Corrected H[#] (Hartree)**	**Corrected G[##] (Hartree)**
NO_3^-	-280.365347	-280.394235	-280.3837588	-280.0132436	-279.9948318	-280.0237198
HNO_3	-280.881852	-280.911948	-280.9133689	-280.5411463	-280.5096294	-280.5397254
$HNO_3*NO_3^-$	-561.294307	-561.339162	-561.3435736	-560.6012692	-560.5520026	-560.5968576
$(HNO_3)_2*NO_3^-$	-842.207219	-842.266757	-842.2913135	-841.1773358	-841.0932413	-841.1527793
2NP	-511.881214	-511.922122	-511.9990494	-511.2075513	-511.089716	-511.130624
3NP	-511.874976	-511.917094	-511.9924178	-511.2019985	-511.0845567	-511.1266747

4NP	-511.877194	-511.919183	-511.9946468	-511.203526	-511.0860732	-511.1280622
2NP-NO_3^-	-792.27822	-792.334072	-792.4157378	-791.2558511	-791.1183333	-791.1741853
3NP-NO_3^-	-792.28715	-792.343325	-792.4247313	-791.2647183	-791.127137	-791.183312
4NP-NO_3^-	-792.290488	-792.347336	-792.4280603	-791.2669952	-791.1294229	-791.1862709
2NP-HNO_3*NO_3^-	-1073.192841	-1073.263559	-1073.363694	-1071.832293	-1071.66144	-1071.732158
3NP-HNO_3*NO_3^-	-1073.203123	-1073.27359	-1073.373833	-1071.843302	-1071.672592	-1071.743059
4NP-HNO_3*NO_3^-	-1073.205339	-1073.276959	-1073.376378	-1071.844402	-1071.673362	-1071.744982
$C_6H_{11}O_3NO$-NO_3^-	-870.978146	-871.040138	-871.1911338	-869.9308992	-869.7179114	-869.7799034
$C_6H_{11}O_3NO_2$-NO_3^-	-946.164376	-946.227655	-946.3839127	-945.0238814	-944.8043447	-944.8676237
$C_6H_{11}O_2NO_2$-NO_3^-	-871.032721	-871.094237	-871.2479662	-869.9809504	-869.7657053	-869.8272213
$C_6H_9O_4NO$-NO_3^-	-944.990475	-945.058272	-945.1822076	-943.8318598	-943.6401272	-943.7079242
$C_6H_9O_4NO_2$-NO_3^-	-1020.176515	-1020.24637	-1020.374632	-1018.923854	-1018.725737	-1018.795592
$C_6H_9O_3NO_2$-NO_3^-	-945.044641	-945.113245	-945.238204	-943.8809658	-943.6874028	-943.7560068
$C_6H_9O_6NO$-NO_3^-	-1095.383421	-1095.456903	-1095.586165	-1094.04758	-1093.844836	-1093.918318
$C_6H_9O_6NO_2$-NO_3^-	-1170.56686	-1170.641909	-1170.776212	-1169.13713	-1168.927778	-1169.002827
$C_6H_9O_5NO_2$-NO_3^-	-1095.425116	-1095.498069	-1095.62969	-1094.085485	-1093.880911	-1093.953864
$C_6H_9O_6^aNO$-NO_3^-	-1095.385908	-1095.456155	-1095.589117	-1094.050765	-1093.847556	-1093.917803
C_6H_9O6$^aNO_2$-NO_3^-	-1170.57183	-1170.644036	-1170.781436	-1169.142746	-1168.93314	-1169.005346
$C_6H_9O_5^aNO_2$-NO_3^-	-1095.440415	-1095.509514	-1095.645741	-1094.10108	-1093.895754	-1093.964853
$C_6H_{11}O_3NO$-HNO_3*NO_3^-	-1151.897102	-1151.974531	-1152.14258	-1150.512004	-1150.266526	-1150.343955
$C_6H_{11}O_3NO_2$-HNO_3*NO_3^-	-1227.0815	-1227.159478	-1227.333445	-1225.602001	-1225.350056	-1225.428034
$C_6H_{11}O_2NO_2$-HNO_3*NO_3^-	-1151.950211	-1152.026032	-1152.197932	-1150.559782	-1150.312061	-1150.387882
$C_6H_9O_4NO$-HNO_3*NO_3^-	-1225.910659	-1225.992096	-1226.13476	-1224.413807	-1224.189705	-1224.271142
$C_6H_9O_4NO_2$-HNO_3*NO_3^-	-1301.096299	-1301.179348	-1301.326869	-1299.505504	-1299.274934	-1299.357983
$C_6H_9O_3NO_2$-HNO_3*NO_3^-	-1225.964381	-1226.045142	-1226.190523	-1224.462618	-1224.236476	-1224.317237
$C_6H_9O_6NO$-HNO_3*NO_3^-	-1376.298621	-1376.387166	-1376.533972	-1374.625007	-1374.389656	-1374.478201
$C_6H_9O_6NO_2$-HNO_3*NO_3^-	-1451.482988	-1451.571734	-1451.725242	-1449.715589	-1449.473334	-1449.56208

#Corrected H = (Sum of electronic and thermal enthalpies(H))$_{DFT}$ - (Electronic Energy$_{DFT}$(E) - Single-point Electronic Energy$_{DLPNO\text{-}CCSD(T)}$(E(CCSD(T)))

##Corrected G = (Sum of electronic and thermal Free Energies(G))$_{DFT}$ - (Electronic Energy$_{DFT}$(E) - Single-point Electronic Energy$_{DLPNO\text{-}CCSD(T)}$(E(CCSD(T)))

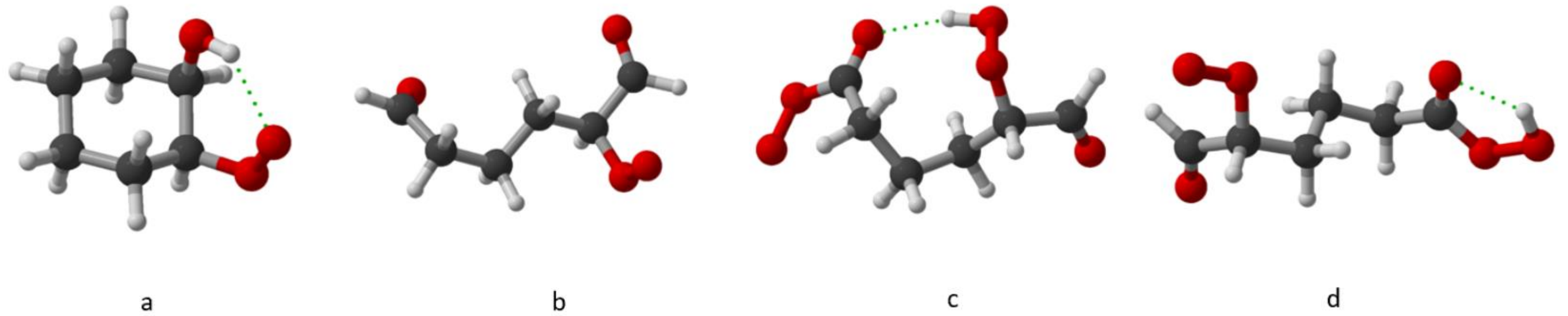

Figure S3. The optimized minimum energy geometries of a) $C_6H_{11}O_3$, b) $C_6H_9O_4$ c) $C_6H_9O_6$ and d) $C_6H_9O_6^a$ peroxy radicals at ωB97X-D/ aug-cc-pVTZ level of theory. Color coding: black is carbon, red is oxygen, white is hydrogen, and green dashed line represents hydrogen bonding.

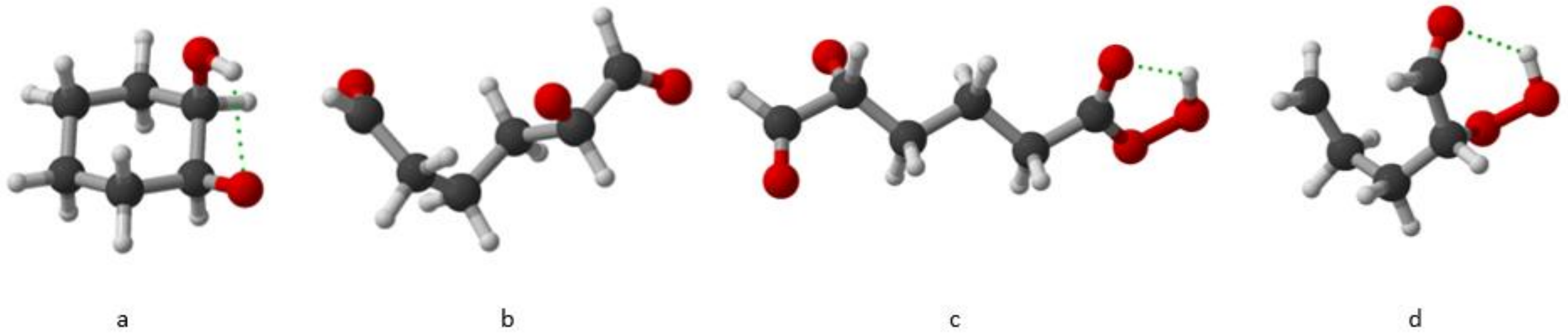

Figure S4. The optimized minimum energy geometries of a) $C_6H_{11}O_2$, b) $C_6H_9O_3$, c) $C_6H_9O_5^a$ and d) $C_5H_9O_3$ radicals at ωB97X-D/ aug-cc-pVTZ level of theory. Color coding: black is carbon, red is oxygen, white is hydrogen, and green dashed line represents hydrogen bonding.

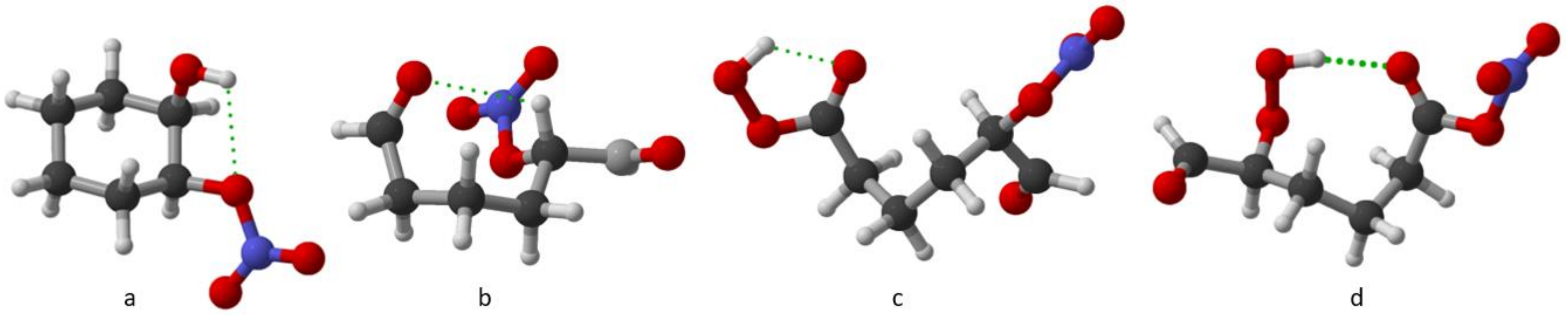

Figure S5. The optimized minimum energy geometries of organic nitrates : a) $C_6H_{11}O_2NO_2$, b) $C_6H_9O_3NO_2$ and c) $C_6H_9O_5^aNO_2$ d) $C_6H_9O_5NO_2$ at ωB97X-D/ aug-cc-pVTZ level of theory. Color coding: black is carbon, red is oxygen, blue is nitrogen, white is hydrogen, and green dashed line represents hydrogen bonding.

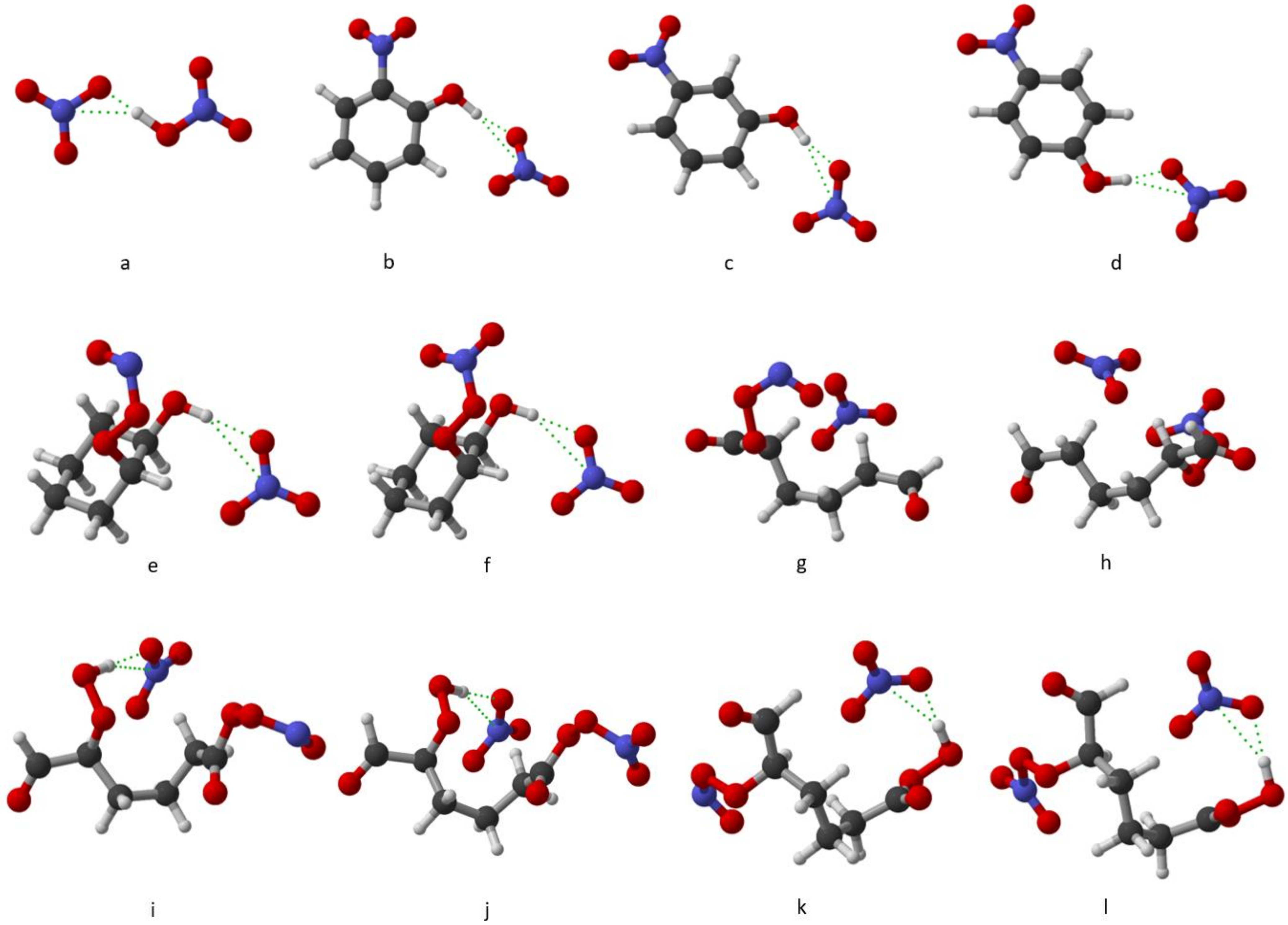

Figure S6. The optimized minimum energy geometries of a) $HNO_3*NO_3^-$, b) 2-Nitrophenol-NO_3^-, c) 3-Nitrophenol-NO_3^-, d) 4-Nitrophenol-NO_3^-, e) $C_6H_{11}O_3NO$-NO_3^-, f) $C_6H_{11}O_3NO_2$-NO_3^-, g) $C_6H_9O_4NO$-NO_3^-, h) $C_6H_9O_4NO_2$-NO_3^- i) $C_6H_9O_6NO$-NO_3^-, j) $C_6H_9O_6NO_2$-NO_3^-, k) $C_6H_9O_6{}^aNO$-NO_3^- and l) $C_6H_9O_6{}^aNO_2$-NO_3^- at ωB97X-D/ aug-cc-pVTZ level of theory. Color coding: black is carbon, red is oxygen, blue is nitrogen, white is hydrogen, and green dashed line represents hydrogen bonding.

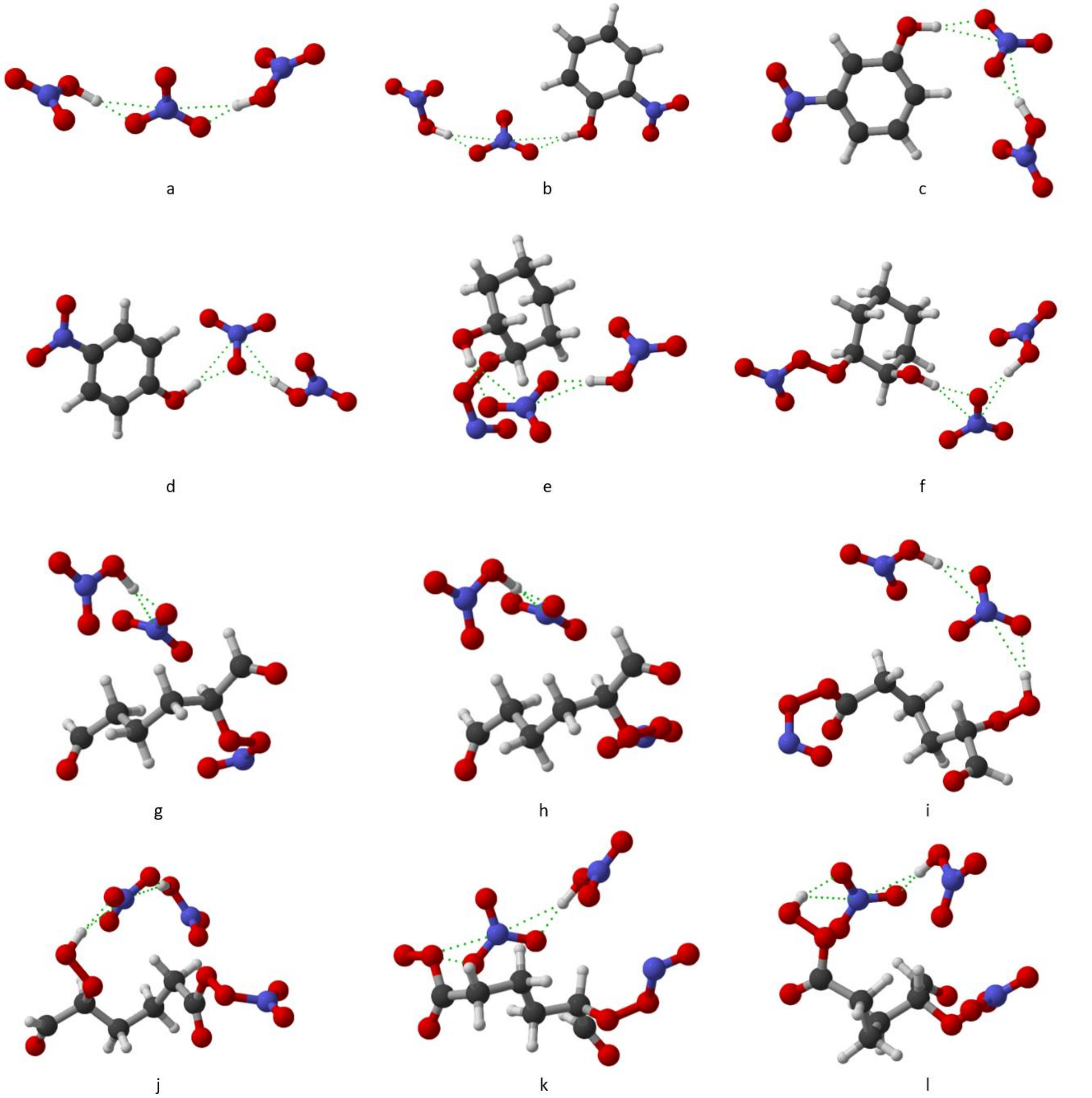

Figure S7. The optimized minimum energy geometries of a) HNO_3-HNO_3*NO_3^-, b) 2-Nitrophenol-HNO_3*NO_3^-, c) 3-Nitrophenol-HNO_3*NO_3^-, d) 4-Nitrophenol-HNO_3*NO_3^-, e) $C_6H_{11}O_3NO$-HNO_3*NO_3^-, f) $C_6H_{11}O_3NO_2$-HNO_3*NO_3^-, g) $C_6H_9O_4NO$-HNO_3*NO_3^-, h) $C_6H_9O_4NO_2$-HNO_3*NO_3^-, i) $C_6H_9O_6NO$-HNO_3*NO_3^-, j) $C_6H_9O_6NO_2$-HNO_3*NO_3^-, k) $C_6H_9O_6{}^{a}NO$-HNO_3*NO_3^-, and l) $C_6H_9O_6{}^{a}NO_2$-HNO_3*NO_3^- at ωB97X-D/ aug-cc-pVTZ level of theory. Color coding: black is carbon, red is oxygen, blue is nitrogen, white is hydrogen, and green dashed line represents hydrogen bonding.

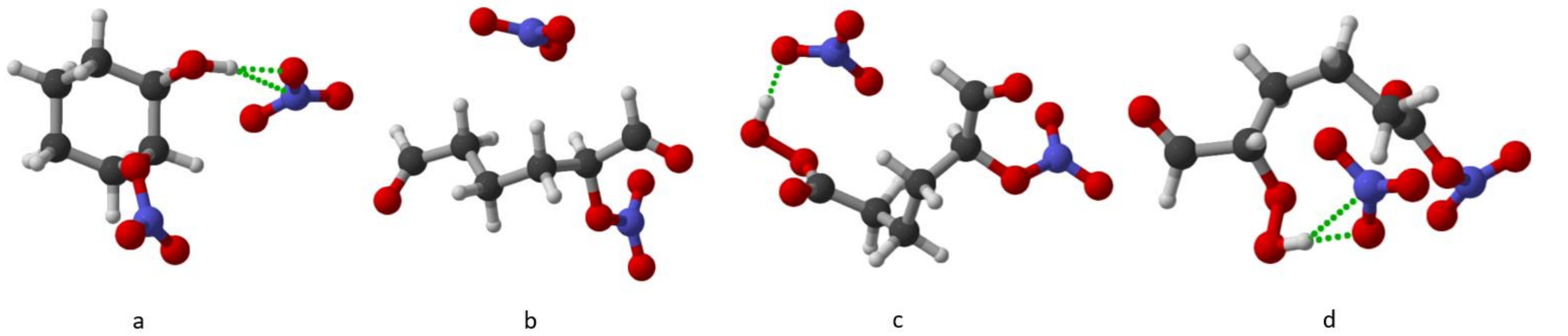

Figure S8. The optimized minimum energy geometries of a) $C_6H_{11}O_2NO_2\text{-}NO_3^-$, b) $C_6H_9O_3NO_2\text{-}NO_3^-$ c) $C_6H_9O_5{}^aNO_2\text{-}NO_3^-$, d) $C_6H_9O_5NO_2\text{-}NO_3^-$ at ωB97X-D/ aug-cc-pVTZ level of theory. Color coding: black is carbon, red is oxygen, blue is nitrogen, white is hydrogen, and green dashed line represents hydrogen bonding.

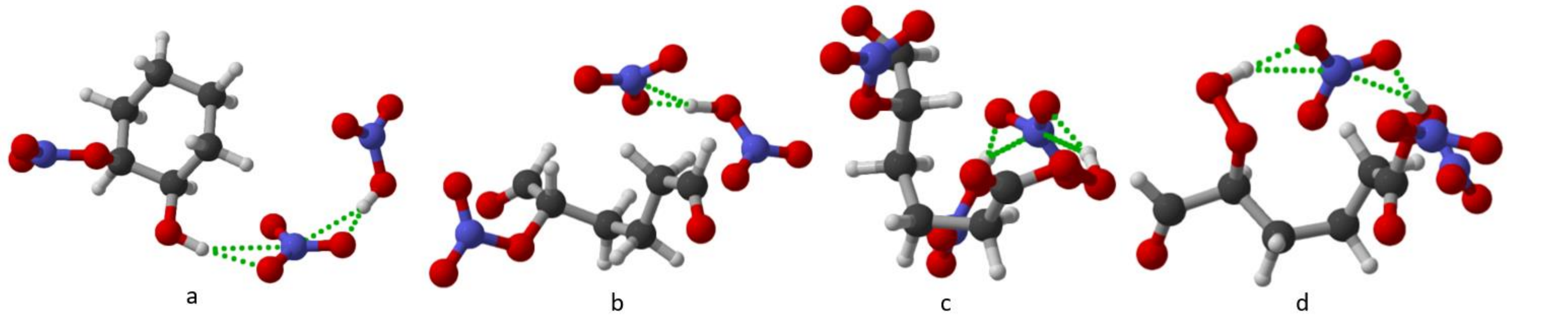

Figure S9. The optimized minimum energy geometries of a) $C_6H_{11}O_2NO_2\text{-}HNO_3{*}NO_3^-$, b) $C_6H_9O_3NO_2\text{-}HNO_3{*}NO_3^-$, c) $C_6H_9O_5{}^aNO_2\text{-}HNO_3{*}NO_3^-$, and d) $C_6H_9O_5NO_2\text{-}HNO_3{*}NO_3^-$ at ωB97X-D/ aug-cc-pVTZ level of theory. Color coding: black is carbon, red is oxygen, blue is nitrogen, white is hydrogen, and green dashed line represents hydrogen bonding.

Section S2 Estimation of effective nitrate-forming rate coefficients using literature-based nitrate yields

The RO_2 + NO reaction is described as a sequential process involving the formation of a chemically activated RO_2-NO intermediate (k, $cm^3\ molecule^{-1}\ s^{-1}$), which can subsequently undergo unimolecular decomposition back to RO_2 + NO (k_1, s^{-1}), dissociation to RO + NO_2 (k_2, s^{-1}) or rearrangement to form organic nitrate $RO\text{-}NO_2$ (k_3, s^{-1}) , as shown in Figure S10.

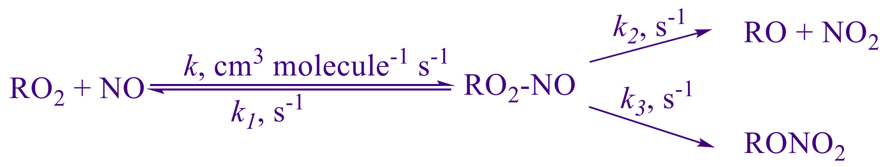

Figure S10. Schematic representation of the RO_2 + NO reaction mechanism, illustrating formation of the chemically activated RO_2-NO intermediate through bimolecular association (k, $cm^3\ molecule^{-1}\ s^{-1}$), followed by unimolecular pathways corresponding to RO_2 + NO back-dissociation (k_1, s^{-1}), RO + NO_2 formation (k_2, s^{-1}) and $RO\text{-}NO_2$ formation (k_3, s^{-1}).

As the nitrate-forming pathway (k_3, s^{-1}) could not be calculated directly in the present study, we used literature-based nitrate yields (Y) reported by Jenkin et al. to provide an approximate estimate of the branching ratios between the possible decomposition channels of the RO_2-NO intermediate.[2] The nitrate yield obtained using this parameterization is given by equation S1:

$$Y = f_a f_b \left(\frac{R^\circ}{1+R^\circ}\right) \qquad \text{(S1)}$$

In the parameterization, f_a accounts for the effect of the substitution pattern of the peroxy radical on nitrate formation, i.e. whether the reacting RO_2 center is primary, secondary, or tertiary. For the RO_2 radicals considered here, the nitrate-forming center is secondary, and therefore $f_a = 1$ is used in all cases. The factor f_b accounts for the influence of substituted functional groups on nitrate yield. For $C_6H_{11}O_3NO$, $f_b = 0.65$ is used because this β-hydroxy peroxy radical contains an OH substituent. For $C_6H_9O_4NO$ and $C_6H_9O_6{}^aNO$, $f_b = 0.3$ assigned here follows β-carbonyl-substituted systems, where this parameterization was originally derived for ketones. Due to the lack of a more specific parameterization for the aldehyde-containing species considered here, its application could constitute a

source of uncertainty. For $C_6H_9O_6NO$ $f_b = 0$ is assigned, following the parameterization which assumes negligible nitrate formation from acyl RO_2 species. The reference branching ratio $R°$ (equation S1) is calculated as

$$R° = \left(\frac{A}{1+(A/B)}\right) F^z \qquad \text{(S2)}$$

where A = 2 x 10^{-22}exp(n_{CON})[M], B = 0.43(T/300)$^{-8}$, F = 0.41, and z = $[1 + (\log_{10}(A/B))^2]^{-1}$. Here, n_{CON} is the number of C, O, and N atoms in the organic group (excluding peroxy oxygens), [M] is the gas number density, and T is the temperature (K). The resulting nitrate yields are summarized in Table S20.

Table S20. Approximate organic nitrate yields (*Y*, %) for the studied RO_2 radicals reacting with NO, calculated using the Jenkin et al. parameterization.

**Species**	**$RONO_2$ Yield (%)**
$C_6H_{11}O_3NO$	14.06 %
$C_6H_9O_4NO$	7.51 %
$C_6H_9O_6^aNO$	8.47 %
$C_6H_9O_6NO$	0.00 %

Using the parameterization the estimated nitrate yield (*Y*), for $C_6H_{11}O_3NO$ was found to be 14.06%. This value falls within the experimental range reported by Teng et al. (2015) for C_6 β-hydroxy peroxy radicals (produced from OH-addition pathway) reacting with NO, where an overall branching ratio of 21 ± 8% was reported for 1-hexene.[2,3] Teng et al. also observed that nitrate yields can vary significantly depending on molecular structure even among similar C_6 systems (e.g., internal alkenes), indicating structural effects on nitrate formation.[3] Consistent with this behavior, application of the Jenkin et al. parameterization to the more oxygenated RO_2 radicals considered here resulted in different (lower than C_6 containing $C_6H_{11}O_3NO$) nitrate yields of ~7 - 8%, arising from functional-group-dependent corrections introduced through the f_b values. Given the limited information available for nitrate yields of highly oxygenated RO_2 systems, these estimates should be regarded as approximate. For the acyl peroxy radical system reacting with NO ($C_6H_9O_6NO$), the applied parameter ($f_b = 0$) predicts negligible nitrate formation based on the general lack of observed acyl nitrate products in systems involving acyl peroxy radicals.[2] This could possibly be explained by rapid CO_2 elimination occurring during rearrangement of the RO_2-NO intermediate toward nitrate formation, thereby competing with completion of the organic nitrate-forming pathway.

Approximate branching ratios for the unimolecular decomposition of RO_2-NO are summarized in Table S21. Within this treatment, branching is evaluated between the RO-NO_2 (*Y* %) and RO + NO_2 ((1 - *Y*) %) formation channels, while the RO_2 + NO back-dissociation pathway is neglected due to its negligible contribution relative to RO + NO_2 formation as indicated by the dissociation rate coefficients reported in Table 3 of the main manuscript and Table S13 of the Supplementary Information.

Table S21. Approximate branching ratios (%) for the unimolecular decomposition of the RO_2-NO intermediate.

**Species**	**RO_2-NO → RO +NO_2 (%)**	**RO_2-NO → RO-NO_2 (%)**
$C_6H_{11}O_3NO$	85.94	14.06
$C_6H_9O_4NO$	92.48	7.51
$C_6H_9O_6^aNO$	91.52	8.47

References

1 M. H. Matus, M. T. Nguyen and D. A. Dixon, *J. Phys. Chem. A*, 2007, **111**, 113–126.
2 M. E. Jenkin, R. Valorso, B. Aumont and A. R. Rickard, *Atmospheric Chemistry and Physics*, 2019, **19**, 7691–7717.
3 A. P. Teng, J. D. Crounse, L. Lee, J. M. St. Clair, R. C. Cohen and P. O. Wennberg, *Atmospheric Chemistry and Physics*, 2015, **15**, 4297–4316.
